# Selective Serotonin Reuptake Inhibitors and Violent Crime: A Cohort Study

**DOI:** 10.1371/journal.pmed.1001875

**Published:** 2015-09-15

**Authors:** Yasmina Molero, Paul Lichtenstein, Johan Zetterqvist, Clara Hellner Gumpert, Seena Fazel

**Affiliations:** 1 Center for Psychiatry Research, Department of Clinical Neuroscience, Karolinska Institutet, Stockholm, Sweden; 2 Department of Medical Epidemiology and Biostatistics, Karolinska Institutet, Stockholm, Sweden; 3 Department of Psychiatry, Warneford Hospital, University of Oxford, Oxford, United Kingdom; Massachusetts General Hospital and Harvard Medical School, Boston, United States of America

## Abstract

**Background:**

Although selective serotonin reuptake inhibitors (SSRIs) are widely prescribed, associations with violence are uncertain.

**Methods and Findings:**

From Swedish national registers we extracted information on 856,493 individuals who were prescribed SSRIs, and subsequent violent crimes during 2006 through 2009. We used stratified Cox regression analyses to compare the rate of violent crime while individuals were prescribed these medications with the rate in the same individuals while not receiving medication. Adjustments were made for other psychotropic medications. Information on all medications was extracted from the Swedish Prescribed Drug Register, with complete national data on all dispensed medications. Information on violent crime convictions was extracted from the Swedish national crime register. Using within-individual models, there was an overall association between SSRIs and violent crime convictions (hazard ratio [HR] = 1.19, 95% CI 1.08–1.32, *p <* 0.001, absolute risk = 1.0%). With age stratification, there was a significant association between SSRIs and violent crime convictions for individuals aged 15 to 24 y (HR = 1.43, 95% CI 1.19–1.73, *p <* 0.001, absolute risk = 3.0%). However, there were no significant associations in those aged 25–34 y (HR = 1.20, 95% CI 0.95–1.52, *p =* 0.125, absolute risk = 1.6%), in those aged 35–44 y (HR = 1.06, 95% CI 0.83–1.35, *p =* 0.666, absolute risk = 1.2%), or in those aged 45 y or older (HR = 1.07, 95% CI 0.84–1.35, *p =* 0.594, absolute risk = 0.3%). Associations in those aged 15 to 24 y were also found for violent crime arrests with preliminary investigations (HR = 1.28, 95% CI 1.16–1.41, *p <* 0.001), non-violent crime convictions (HR = 1.22, 95% CI 1.10–1.34, *p <* 0.001), non-violent crime arrests (HR = 1.13, 95% CI 1.07–1.20, *p <* 0.001), non-fatal injuries from accidents (HR = 1.29, 95% CI 1.22–1.36, *p <* 0.001), and emergency inpatient or outpatient treatment for alcohol intoxication or misuse (HR = 1.98, 95% CI 1.76–2.21, *p <* 0.001). With age and sex stratification, there was a significant association between SSRIs and violent crime convictions for males aged 15 to 24 y (HR = 1.40, 95% CI 1.13–1.73, *p =* 0.002) and females aged 15 to 24 y (HR = 1.75, 95% CI 1.08–2.84, *p =* 0.023). However, there were no significant associations in those aged 25 y or older. One important limitation is that we were unable to fully account for time-varying factors.

**Conclusions:**

The association between SSRIs and violent crime convictions and violent crime arrests varied by age group. The increased risk we found in young people needs validation in other studies.

## Abstract 2

In a registry-based cohort study, Seena Fazel and colleagues test for within-individual associations between SSRI prescription and violent crime.

## Editors' Summary

**Background:**

Antidepressants—drugs that treat depression (unbearable feelings of sadness and despair caused by changes in brain chemistry)—are widely prescribed in many countries. In the US, for example, about one in ten people over 12 years old take antidepressants. The first antidepressants—monoamine oxidase inhibitors and tricyclic antidepressants—were developed in the 1950s. Experts think that both these classes of drugs treat depression by increasing serotonin levels in the brain. Serotonin, which is thought to improve mood, emotion, and sleep, is a neurotransmitter, a chemical that carries messages between nerve cells. However, monoamine oxidase inhibitors and tricyclic antidepressants had many adverse side effects unrelated to their effects on serotonin levels. So, in the late 1980s, a new class of antidepressant drugs was launched known as selective serotonin reuptake inhibitors (SSRIs). After serotonin delivers a message between nerve cells, it is usually reabsorbed by the nerve cells. Fluoxetine (Prozac), paroxetine (Seroxat), and other SSRIs block this “reuptake,” thereby increasing serotonin levels in the brain.

**Why Was This Study Done?:**

SSRIs (which are also used to treat several other mental health conditions) have fewer side effects than the older antidepressants, although they can cause headache, nausea, sleep problems, restlessness, and sexual problems. However, SSRIs are not recommended for use in people under the age of 18 years because there is some evidence that SSRIs increase the risk of self-harm and suicidal thoughts in this age group. Moreover, there is limited and inconclusive evidence linking SSRI use with violent behavior. Because SSRIs are widely prescribed, it is important to clarify this latter issue. In this cohort study—an observational study that follows a group of individuals who are identical with the exception of exposure to a specific factor to determine whether exposure to that factor increases the likelihood of a specific outcome—the researchers investigate the association between violent crime and SSRIs in Sweden.

**What Did the Researchers Do and Find?:**

The researchers extracted information on SSRIs prescribed in Sweden between 2006 and 2009 from the Swedish Prescribed Drug Register and information on convictions for violent crimes for the same period from the Swedish national crime register. They then compared the rate of violent crime while individuals were prescribed SSRIs with the rate of violent crime in the same individuals while not receiving medication. This “within-individual” design accounts for time-invariant factors such as genetic and early environmental factors that might otherwise lead to confounding. In observational studies, participants exposed to a specific factor can also share another unknown characteristic (confounder) that is actually responsible for the outcome of interest. During the study period, about 850,000 individuals (10.8% of the Swedish population) were prescribed SSRIs, and 1% of these individuals were convicted of a violent crime. Using within-individual statistical models, there was a significant but modest overall association (an association unlikely to have occurred by chance) between SSRIs and convictions for violent crime. After adjustment for age, the association between SSRIs and convictions for violent crimes remained significant for individuals (males and females combined or males and females considered separately) aged 15 to 24 years but became non-significant among older individuals.

**What Do These Findings Mean?:**

These findings show an association between SSRIs and violent crime that varies by age group. They cannot, however, prove that taking SSRIs actually causes an increase in violent crime among young people because the analytical approach used does not fully account for time-varying risk factors such as symptom severity or alcohol misuse that might affect an individual’s risk of committing a violent crime (residual confounding). In addition, some people who committed a violent crime might have subsequently taken SSRIs to cope with the anxiety and stress of arrest (reverse causation). The lack of a significant association between SSRIs and violent crime among most people taking SSRIs is reassuring; the association between violent crimes and SSRIs among individuals younger than 25 years is worrying. However, this finding needs confirming in studies with other designs undertaken in other settings. If confirmed, warnings about the increased risk of violent behavior among young people when being treated with SSRIs might be needed. But, note the researchers, it might be inappropriate to restrict the use of SSRIs in this age group because increases in adverse outcomes associated with poorly treated depression, such as suicide, might outweigh the public health benefit accruing from decreases in violence.

**Additional Information.:**

This list of resources contains links that can be accessed when viewing the PDF on a device or via the online version of the article at http://dx.doi.org/10.1371/journal.pmed.1001875.

- The UK National Health Service Choices website provides information about depression (including personal stories) and about SSRIs; a “behind the headlines” article discusses a research article on recent increases in the use of SSRIs across Europe
- The UK Royal College of Psychiatrists provides leaflets on depression and on antidepressants
- Mind, a UK noMind, a UK not-for-profit organization, also provides information about depression (including personal stories) and about antidepressants
- The US National Institute of Mental Health provides information about depression and about antidepressant medications for children and adolescents
- MedlinePlus provides links to additional resources about depression and antidepressants

## Introduction

Selective serotonin reuptake inhibitors (SSRIs) are among the most widely prescribed psychiatric medications in many countries [1–6]. At the same time, concerns about their adverse effects, including suicide and violence, have been widely discussed and remain controversial. Observational and trial data have shown that although SSRIs appear not to elevate the risk for suicidal behaviour in adults, they may increase the risk of suicide ideation in children, adolescents, and young adults. This weak age-related association is consistent across studies [7–11] but inconsistently supported by ecological data [12–15].

Despite a number of legal cases linking SSRIs and violent behaviour [16], empirical research on the association is limited and inconclusive. Ecological studies suggest that increased SSRI prescriptions have been associated with decreases in violent crimes in the US [17] and lethal violence in the Netherlands [18]. In contrast, an expert review of clinical trials concluded that there was an excess of violence in both adults and children on SSRIs compared with placebo [16]. Furthermore, drug safety (or pharmacovigilance) data have shown a disproportionate association between SSRIs and violent behaviours [19] and serious violent acts [20], and an observational study found an association of work-related violence with antidepressant purchases [21]. However, these study designs are limited: findings from ecological data fail to relate the use of SSRIs at the individual level and are liable to be influenced by secular changes, including legislation, reporting of violence, and unaccounted changes in the impact of other risk factors such as drug and alcohol use [15,22]. Pharmacovigilance data are subject to reporting bias, changes in patient awareness about adverse outcomes, confounding by indication, and failure to account for exposure to other medications [23].

Pharmacoepidemiological studies provide one approach to deal with these limitations [12,23]. Our objective was thus to investigate the association between SSRIs and violence outcomes by linking data from Swedish national registers on individual SSRI prescriptions, use of other psychotropic drugs, and violent crimes in a large population-based cohort. We have primarily used a “within-individual” design [24–27], where the risk of violent crime is determined when an individual is taking an SSRI as compared to when the same person is not. Using this design, all time-invariant factors (i.e., genetic factors, all factors before the start of follow-up, and factors that remain constant during follow-up) are accounted for; thus, this design more fully adjusts for unmeasured time-invariant confounding and confounding by indication than other observational designs, but does not account for time-varying factors such as symptom severity. Our null hypothesis was that no associations between SSRI medication and violent outcomes would be demonstrated using a within-individual design, including in different age groups.

## Methods

In the total population of Sweden aged 15 y or older in 2006 (*n* = 7,917,854) and residing in Sweden during follow-up (January 1, 2006, to December 31, 2009), we identified 856,493 individuals who were prescribed SSRI treatment. Information on individuals receiving SSRI treatment was collected from Swedish population-based registers with national coverage, and registers were linked using each individual’s unique identification number. The project was approved by the ethics committee at Karolinska Institutet (2005/4:5).

### Measures

#### SSRI treatment

Information on medication and the date prescriptions were dispensed was extracted from the Swedish Prescribed Drug Register, with complete national data on all prescribed and dispensed medical drugs from all pharmacies in Sweden since July 2005 [28]. A previous comparison between post-mortem toxicology and SSRI purchases in the Swedish Prescribed Drug Register indicated good medication compliance [29].

In our initial analysis, we included all individuals with dispensed SSRI prescriptions. However, as prescriptions are typically restricted to at most 3 mo and we wanted to restrict the sample to those adherent to SSRIs, individuals with a single SSRI prescription within a 6-mo period were excluded from stratified and sensitivity analyses as no assumptions could be made about their medication adherence. A separate analysis was also carried out including only individuals with a single dispensed prescription. A treatment period was thus defined as a series of SSRI prescriptions with no more than 6 mo between two consecutive prescriptions. The start of a treatment period was defined as the date an SSRI prescription was first dispensed during our follow-up. The end of a treatment period was defined as the date that the last SSRI prescription in that treatment period was dispensed. Periods of more than 6 mo between prescriptions were considered non-treatment periods. A new treatment period was considered to have started at the first date of the next series of consecutive prescriptions (see S1 Methods for details on SSRI medications). For individuals with a single prescription, the start of their treatment period was defined as the date their prescription was dispensed, and the end of that treatment period was defined as 14 d after the prescription was dispensed.

#### Other psychotropic medications

Adjustments were made for concurrent psychotropic medications other than SSRIs, which included antipsychotics, hypnotics, sedatives, anxiolytics, drugs used in addictive disorders, mood stabilisers, antiepileptics, and antidepressant medications other than SSRIs (venlafaxine, duloxetine, tricyclics, heterocyclics, mirtazapine, non-selective monoamine oxidase inhibitors, moclobemide, and bupropion). Treatment periods were defined in the same manner as SSRI treatment periods (see S1 Methods for details).

#### Violent crimes

Information on convictions for violent crimes for individuals aged 15 y and older (the age of criminal responsibility) was extracted from the Swedish national crime register. Violent crimes were defined as crimes against persons as per previous work [30], and included attempted, completed, and aggravated forms of homicide, manslaughter, unlawful threats, harassment, robbery, arson, assault, assault on an official, kidnapping, stalking, coercion, and all sexual offences (see S1 Methods for more details).

#### Alternative outcomes

Examinations of individual SSRIs and alternative outcomes were also carried out, including (1) convictions for substance-related crimes, (2) convictions for non-violent crimes, (3) arrests with preliminary investigations (hereafter “arrests”, as distinct from convictions; described as “suspicions” in the Swedish crime register) for violent crimes, (4) arrests for substance-related crimes, (5) arrests for non-violent crimes, (6) non-fatal injuries (hospitalisations) from accidents; (7) emergency inpatient or outpatient treatment for alcohol intoxication or misuse, (8) and psychiatric hospitalisations (see S1 Methods for details on alternative outcomes).

### Statistical Analyses

Individuals were followed from January 1, 2006, to December 31, 2009, and follow-up was adjusted for migration, periods in prison or institutional youth care, hospitalisation, and death through linkage to the Swedish migration, prison, patient, and cause of death registers. Unobservable time, i.e., time abroad, in prison, or in hospital, was removed (truncated) from the follow-up time. Time after hospital discharge, release from prison, or immigration was added to the observable cohort again.

A between-individual Cox proportional hazards regression compared the average rate of violent crime convictions during SSRI medication with the rate during non-medication for all individuals. In this analysis, follow-up period was split into the period before the first outcome, periods between outcomes, and the period after the last outcome. Time at risk was measured from the start of each period, and medication was used as a time-varying covariate. Robust standard errors were calculated to account for correlations between periods within the same individual. This analysis was adjusted for sex and age.

The principal analyses were within-individual stratified Cox proportional hazards regressions, with each individual entering as a separate stratum in the analysis and serving as his/her own control. The obtained hazard ratio (HR) is thus adjusted for (i.e., stratified by) all potential time-invariant confounders within each individual. To adjust for age, which is a time-varying potential confounder, age was added to the model as a time-varying covariate, with one factor for each whole year. In the within-individual stratified Cox proportional hazards regression, only individuals who changed medication status contributed directly to the estimate. All other individuals contributed indirectly through the estimates of other covariates. Since the covariates in the within-individual stratified Cox proportional hazards regression were time-varying, we did not test for the proportional hazards assumption. More information on this approach is provided in [31]; this approach has been applied in studies of attention deficit hyperactivity disorder medication, antipsychotics, and mood stabilisers [24–27]. To ensure that outcomes were measured appropriately, all crimes were included from the date of perpetration (rather than conviction), and those with uncertain date of perpetration were excluded from the analyses, resulting in the exclusion of 1.3% (1,241) of violent crime convictions, 1.0% (9,108) of non-violent crime convictions, and 1.8% (5,187) of substance-related convictions during the period from 2006 to 2009.

To test for confounding by other psychotropic medications, we first adjusted for concurrent exposure to other psychotropic medications as a time-varying covariate. Then we excluded individuals with other psychotropic medications during follow-up from the within-individual stratified Cox proportional hazards regression. Analyses were also stratified by sex, by age (from age 15 y, the age of criminal responsibility, in 10-y bands [32] up to age 44 y; the age bands for ages 45 y and over were combined as event rates were low), and by type of SSRI medication (fluoxetine, citalopram, paroxetine, sertraline, or escitalopram).

To estimate cumulative exposure to SSRIs, the defined daily dose (DDD) of SSRI medication [33] was calculated through summing dispensed medication and then dividing the sum by the number of days in the treatment period. DDDs were categorised into four groups; (1) no exposure, (2) low SSRI exposure (<1 DDD/day), (3) moderate SSRI exposure (1–2 DDD/day), and (4) high SSRI exposure (>2 DDD/day).

#### Sensitivity analyses

In sensitivity analyses, within-individual stratified Cox proportional hazards regressions were carried out with the following alternative outcomes: convictions for non-violent crimes, convictions for substance-related crimes, arrests for violent crimes, arrests for non-violent crimes, arrests for substance-related crimes, non-fatal injuries from accidents, emergency treatment for alcohol intoxication or misuse, and psychiatric hospitalisations. Furthermore, each SSRI medication was analysed separately, and periods of using of two or more SSRI medications were excluded to adjust for switching effects between SSRI medications. Furthermore, all SSRIs were entered in the same model as covariates to adjust for concurrent use of other SSRIs. Analyses were also stratified by type of SSRI medication with violent crime arrests as an alternative outcome. Additionally, other antidepressants (venlafaxine, duloxetine, tricyclics, heterocyclics, mirtazapine, moclobemide, and bupropion) were used as an alternative exposure for violent crime convictions. Further sensitivity analyses were carried out to test for non-specific treatment effects where diuretics were used as an alternative exposure for violent crime convictions to test the model.

For individuals who started SSRI treatment after being convicted of a violent crime, the number of days between the date of committing the crime and the start of SSRI treatment was calculated. To exclude the possibility of reverse causation, i.e., if committing a violent crime increased the probability of subsequent SSRI treatment, new within-individual stratified Cox proportional hazards regressions were carried out excluding from the analysis all individuals who received SSRI treatment within 7, 14, 30, or 60 d after committing a violent crime.

Finally, the robustness of results was tested by undertaking four alternative analyses. First, a conditional Poisson regression examined how changes in medication exposure were associated with changes in violent crime convictions within the same person, thus adjusting for time-invariant confounders. Second, we repeated the main models with different definitions of a treatment period: (1) a series of SSRI prescriptions with no more than 3 mo between two consecutive prescriptions and (2) a series of SSRI prescriptions with no more than 4 mo between two consecutive prescriptions. Third, we tested for delayed onset of action of SSRIs by setting the first day of the treatment period to 8 wk after the date of the first dispensed prescription. Fourth, we tested for SSRI discontinuation effects by extending the end of the treatment period to 3 wk and 12 wk after the date that the last SSRI prescription in a treatment period was dispensed.

SAS version 9.4 (SAS Institute) was used for all analyses, except for the conditional Poisson regression, for which STATA 13.1 (StataCorp) was used. For SAS, software function “proc phreg” was used for both stratified and marginal Cox regressions, and for STATA, software function “xtpoisson” was used for the conditional Poisson regression. STROBE guidelines were followed (S1 STROBE).

## Results

### Sample Description

Of 7,917,854 individuals in the general population investigated (individuals in Sweden aged 15 y or older in 2006), 856,493 (10.8%) were prescribed SSRIs during the time period 2006–2009, or 14.1% of all women and 7.5% of all men in the investigated population (see Table 1 for background characteristics). Of those prescribed SSRIs, 9.9% were aged 15–24 y, 12.7% were aged 25–34 y, 16.5% were aged 35–44 y, 15.6% were aged 45–54 y, 15.5% were aged 55–64 y, and 29.7% were aged 65 y or over at baseline in 2006. In the SSRI cohort, 8,377 individuals (1.0%) were convicted of a violent crime during the period 2006–2009. Among the individuals who were prescribed SSRI treatment, 65,862 individuals were prescribed fluoxetine, 389,857 citalopram, 46,615 paroxetine, 215,873 sertraline, 1,198 fluvoxamine, and 84,934 escitalopram.

**Table 1:** Characteristics at baseline and during follow-up for SSRI-medicated and non-medicated individuals in a population sample in Sweden 2006–2009.

Characteristic	Non-Medicated	SSRI-Medicated^ǂ^
	89.2% (7,061,361)	10.8% (856,493)
***Characteristics at baseline***		
**Sex**		
Women	48.8% (3,443,970)	65.9% (564,278)
Men	51.2% (3,617,391)	34.1% (292,215)
**Age**		
15 to 24 y	20.4% (1,439,183)	9.9% (84,647)
25 to 34 y	14.7% (1,039,843)	12.7% (108,928)
35 to 44 y	16.2% (1,144,303)	16.5% (141,375)
45 to 54 y	14.6% (1,029,305)	15.6% (133,996)
55 to 64 y	15.3% (1,082,914)	15.5% (132,995)
65 y and over	18.8% (1,325,813)	29.7% (254,552)
**Lifetime psychiatric diagnoses**		
Psychotic disorder	0.9% (63,242)	3.3% (27,838)
Mood disorder	2.0% (143,910)	23.2% (198,366)
Anxiety, dissociative, stress-related, or somatoform disorder	2.6% (180,735)	20.3% (173,665)
Eating disorder	0.2% (10,965)	1.2% (10,414)
Substance (alcohol and drug) use disorder	2.7% (193,164)	9.1% (77,746)
***Characteristics during follow-up***		
**Other psychotropic medications**		
Antipsychotic	0.3% (24,515)	0.9% (7,998)
Hypnotic, sedative, or anxiolytic	11.5% (814,717)	37.4% (319,987)
Drug used in addictive disorders	1.1% (74,736)	2.5% (21,494)
Mood stabiliser	0.8% (58,519)	2.5% (21,151)
Antiepileptic medication	1.0% (74,474)	3.4% (28,722)
Venlafaxine	0.5% (32,911)	3.0% (25,906)
Duloxetine	0.3% (18,135)	2.2% (18,871)
Tricyclic	1.7% (116,963)	4.7% (40,272)
Heterocyclic	0.2% (11,622)	2.3% (19,770)
Mirtazapine	1.4% (95,223)	14.0% (119,757)
Non-selective monoamine oxidase inhibitor	<0.1% (133)	<0.1% (86)
Moclobemide	<0.1% (1,669)	0.2% (1,548)
Bupropion	0.5% (37,116)	2.1% (17,952)
Any psychotropic medicine other than SSRIs	19.3% (1,360,733)	75.1% (643,514)
**Outcomes**		
Convicted of a violent crime	0.6% (40,384)	1.0% (8,377)
Convicted of a non-violent crime	2.5% (178,622)	3.3% (28,358)
Convicted of a substance-related crime	1.0% (67,044)	1.7% (14,625)
Arrested for a violent crime	1.9% (135,495)	3.0% (25,624)
Arrested for a non-violent crime	3.8% (270,203)	5.6% (47,244)
Arrested for a substance-related crime	1.2% (82,723)	2.1% (17,787)
Non-fatal injury from an accident	14.0% (991,329)	21.0% (179,734)
Emergency treatment for alcohol intoxication or misuse	0.9% (66,659)	2.8% (24,141)
Psychiatric hospitalisation	1.2% (86,912)	9.5% (81,406)
**Periods of SSRI exposure** *		
No SSRI exposure		49.4% (10,540,144)
Low SSRI exposure (<1 DDD/day)		16.2% (3,466,382)
Moderate SSRI exposure (1–2 DDD/day)		24.9% (5,327,339)
High SSRI exposure (>2 DDD/day)		9.5% (2,026,178)

Data are given as percent (*n*). See S1 Methods for details on characteristics at baseline and during follow-up.

^**ǂ**^Including all individuals with dispensed SSRI prescriptions.

*Numbers are periods of SSRI exposure rather than individuals.

### Main Analyses

Within-individual Cox proportional hazards analyses were carried out to compare violent crime rates within the same individuals during periods when they were on medication compared to periods when they were not, and the results showed an increased risk of violent crime conviction during medicated periods (HR = 1.19, 95% CI 1.08–1.32, *p* < 0.001; Table 2). The estimated hazard did not materially change when we adjusted for concurrent psychotropic medications (HR = 1.22, 95% CI 1.11–1.32, *p <* 0.001), nor when we excluded individuals with only one dispensed prescription (HR = 1.22, 95% CI 1.10–1.35, *p <* 0.001). Additionally, when we excluded all individuals who had received other psychotropic medications during follow-up from the analysis, the estimated hazard was similar (HR = 1.20, 95% CI 1.04–1.38, *p =* 0.014). The between-individual Cox proportional hazards analysis also demonstrated an association between SSRI prescriptions and being convicted of a violent crime (HR = 2.66, 95% CI 2.54–2.78, *p <* 0.001) when comparing individuals on SSRIs to individuals who were not taking SSRIs.

**Table 2:** Violent crime convictions in 2006–2009 in individuals treated with SSRI medication as compared to non-medicated individuals, and comparing treatment to non-treatment periods within the same person.

Analysis	HR (95% CI)	*p*-Value	Number of Events
**Within-individual stratified Cox proportional hazards regression**			
All individuals^ǂ^	1.19 (1.08–1.32)	<0.001	11,225
Excluding individuals with only one dispensed prescription	1.22 (1.10–1.35)	<0.001	8,592
Including only individuals with only one dispensed prescription	0.73 (0.45–1.17)	0.193	3,949
**Analysis adjusted for concurrent psychotropic medication**			
All individuals adjusted for other psychotropic medications	1.22 (1.11–1.32)	<0.001	11,225
Excluding individuals with other psychotropic medications during follow-up	1.20 (1.04–1.38)	0.014	5,949
**Between-individual Cox proportional hazards regression**			
All individuals^ǂ^	2.66 (2.54–2.78)	<0.001	62,342

^**ǂ**^Including all individuals with dispensed SSRI prescriptions.

The analyses were then stratified by sex and age band (Table 3). This demonstrated an increased risk of violent crime conviction for those aged 15 to 24 y (HR = 1.43, 95% CI 1.19–1.73, *p <* 0.001) but not for the other age bands investigated (25–34 y, 35–44 y, and 45 y and older). When stratified by sex and age, associations were significant for both genders in the age group 15 to 24 y (HR = 1.40, 95% CI 1.13–1.73, *p =* 0.002, and HR = 1.75, 95% CI 1.08–2.84, *p =* 0.023, for males and females respectively). Next, the role of cumulative SSRI exposure was examined using DDDs. The results showed that low SSRI exposure was associated with an increased risk of being convicted of a violent crime as compared to periods of non-exposure (HR = 1.27, 95% CI 1.10–1.47, *p =* 0.001). However, no significant association with violent crime conviction was found for periods of moderate or high SSRI exposure (Table 3).

**Table 3:** Violent crime convictions in 2006–2009 in individuals treated with SSRI medication compared to non-treatment periods in the same person stratified by sex, age, dose, and medication type using stratified Cox regression models.

Characteristic	HR (95% CI)	*p*-Value	Number of Events	Absolute Risk, Percent (*n*)
**Sex**				
Males	1.22 (1.09–1.37)	<0.001	6,905	2.1 (5,020)
Females	1.21 (0.94–1.56)	0.141	1,687	0.3 (1,418)
**Age**				
15 to 24 y	1.43 (1.19–1.73)	<0.001	2,798	3.0 (2,081)
25 to 34 y	1.20 (0.95–1.52)	0.125	1,966	1.6 (1,454)
35 to 44 y	1.06 (0.83–1.35)	0.666	1,905	1.2 (1,457)
45 y and over	1.07 (0.84–1.35)	0.594	1,918	0.3 (1,446)
**Sex and age: males**				
15 to 24 y	1.40 (1.13–1.73)	0.002	2,169	6.6 (1,540)
25 to 34 y	1.16 (0.90–1.49)	0.265	1,629	3.6 (1,173)
35 to 44 y	1.11 (0.85–1.46)	0.428	1,538	2.8 (1,133)
45 y and over	0.97 (0.75–1.25)	0.792	1,566	0.8 (1,174)
**Sex and age: females**				
15 to 24 y	1.75 (1.08–2.84)	0.023	629	1.2 (541)
25 to 34 y	1.67 (0.88–3.18)	0.119	337	0.5 (281)
35 to 44 y	0.76 (0.42–1.41)	0.388	367	0.4 (324)
45 y and over	1.80 (0.97–3.32)	0.062	352	0.1 (272)
**Cumulative SSRI exposure**				
Low SSRI exposure	1.27 (1.10–1.47)	0.001	896	n/a
Moderate SSRI exposure	1.11 (0.96–1.28)	0.163	1,038	n/a
High SSRI exposure	1.06 (0.88–1.29)	0.528	562	n/a
**Type of SSRI medication**				
Fluoxetine	1.16 (0.88–1.52)	0.287	1,276	1.4 (916)
Citalopram	1.14 (0.96–1.36)	0.130	3,088	0.6 (2,292)
Paroxetine	1.24 (0.87–1.77)	0.227	784	1.2 (577)
Sertraline	1.22 (1.02–1.46)	0.031	3,003	1.0 (2,241)
Escitalopram	1.02 (0.77–1.34)	0.904	1,446	1.2 (1,051)

Analyses excluded individuals with only one dispensed prescription. There were too few individuals for separate analyses of fluvoxamine.

n/a, not applicable.

### Sensitivity Analyses

In sensitivity analyses, our results showed some differences for individual SSRIs; there was a significantly higher hazard for violent crime conviction in individuals prescribed sertraline (Table 3) and for violent crime conviction in individuals prescribed citalopram and sertraline after eliminating periods of concurrent use of two different SSRIs, thus adjusting for switching effects between SSRIs (Table 4). For violent crime arrests, the increased association with citalopram remains (Table 4).

**Table 4:** Sensitivity analyses: rates of different adverse outcomes in individuals treated with SSRI medication and other antidepressants compared to non-treatment periods in the same person using stratified Cox regression models.

Outcome/Exposure	HR (95% CI)	*p*-Value	Number of Events
**Alternative outcomes**			
Convicted of a non-violent crime	1.10 (1.05–1.15)	<0.001	51,888
Convicted of a substance-related crime	0.99 (0.93–1.06)	0.825	17,922
Arrested for a violent crime	1.13 (1.08–1.18)	<0.001	33,556
Arrested for a non-violent crime	1.05 (1.02–1.08)	<0.001	85,645
Arrested for a substance-related crime	0.99 (0.94–1.05)	0.772	23,919
Non-fatal injury from an accident	1.20 (1.18–1.23)	<0.001	241,900
Emergency treatment for alcohol intoxication or misuse	1.06 (1.03–1.09)	<0.001	70,262
Psychiatric hospitalisation^ǂ^	0.96 (0.93–0.99)	0.012	200,999
**Type of SSRI medication, with violent crime convictions as outcome, excluding periods of concurrent use of other SSRI medications**			
Fluoxetine	1.25 (0.95–1.65)	0.115	1,276
Citalopram	1.20 (1.00–1.43)	0.048	3,088
Paroxetine	1.23 (0.87–1.75)	0.250	784
Sertraline	1.22 (1.01–1.46)	0.037	3,003
Escitalopram	1.07 (0.81–1.44)	0.613	1,446
**Type of SSRI medication, with violent crime convictions as outcome, adjusted for other SSRI medications**			
Fluoxetine	1.17 (0.89–1.53)	0.255	1,276
Citalopram	1.16 (0.97–1.37)	0.102	3,088
Paroxetine	1.26 (0.89–1.79)	0.194	784
Sertraline	1.23 (1.03–1.48)	0.023	3,003
Escitalopram	1.07 (0.82–1.42)	0.604	1,446
**Type of SSRI medication, with violent crime arrests as outcome**			
Fluoxetine	1.03 (0.91–1.17)	0.612	4,836
Citalopram	1.15 (1.06–1.24)	0.001	12,595
Paroxetine	1.24 (1.06–1.24)	0.007	2,840
Sertraline	1.08 (0.99–1.17)	0.057	11,976
Escitalopram	1.06 (0.93–1.20)	0.383	5,097
**Other antidepressants and medications, with violent crime convictions as outcome**			
Venlafaxine	1.39 (1.10–1.78)	0.007	1,843
Duloxetine	1.26 (0.88–1.81)	0.200	761
Tricyclics	1.05 (0.71–1.55)	0.821	804
Heterocyclics	1.64 (0.92–2.95)	0.097	330
Mirtazapine	0.71 (0.59–0.88)	0.001	3,258
Moclobemide	1.70 (0.36–7.91)	0.502	46
Bupropion	0.42 (0.14–1.24)	0.118	211
Diuretics	0.80 (0.67–0.95)	0.012	3,450

Analyses excluded individuals with only one dispensed prescription. There were too few individuals for separate analyses of fluvoxamine.

^**ǂ**^Adjusted model: please see S1 Methods for more details.

In further analyses, the relationship between SSRI treatment and other outcomes was examined (Table 4), and the results showed an increased risk of violent crime arrests, non-violent crime convictions, and non-violent crime arrests with SSRI treatment. Furthermore, an increased risk of non-fatal injuries from accidents was found (HR = 1.20, 95% CI 1.18–1.23, *p <* 0.001). The possible role of alcohol misuse as a time-varying confounder was tested by using emergency inpatient and or outpatient treatment for alcohol intoxication or misuse as an outcome, showing an increased risk during times of medication (HR = 1.06, 95% CI 1.03–1.09, *p <* 0.001). The risk of hospitalisation for psychiatric care was also examined, showing a slightly decreased risk with SSRI treatment (HR = 0.96, 95% CI 0.93–0.99, *p =* 0.012). When we investigated other antidepressant classes, we found a significant association between medication use and violent crime conviction for individuals prescribed venlafaxine. The risk of being convicted of a violent crime was reduced when on mirtazapine. Finally, an inverse association between violent crime conviction and diuretics was found using the within-individual model (HR = 0.80, 95% CI 0.67–0.95, *p =* 0.012).

When all analyses were stratified by age (S1 Table), the increased risk of being convicted of a violent crime remained in individuals aged 15 to 24 y after adjustment for concurrent psychotropic medications (HR = 1.45, 95% CI 1.21–1.74, *p <* 0.001). Results also showed that low SSRI exposure was associated with an increased risk of being convicted of a violent crime in this age band only (HR = 1.62, 95% CI 1.23–2.13, *p <* 0.002). Furthermore, significant associations were shown for violent crime arrests and non-violent crime arrests and convictions for individuals aged 15 to 24 y, and also for individuals aged 25 to 34 y, although associations were weaker in the latter age band. The increased risk of non-fatal injuries from accidents remained significant for all ages. Results also showed that individuals aged 15–24, 25–34, and 35–44 y had an increased risk of emergency inpatient or outpatient treatment for alcohol intoxication or misuse (HR = 1.98, 95% CI 1.76–2.21, *p <* 0.001; HR = 1.33, 95% CI 1.21–1.46, *p <* 0.001; HR = 1.08, 95% CI 1.01–1.14, *p =* 0.015, respectively). However, individuals aged 45 y and older showed a slightly decreased risk of emergency inpatient or outpatient treatment for alcohol intoxication or misuse (HR = 0.96, 95% CI 0.93–0.99, *p =* 0.028).

To test whether individuals who had been dispensed only one prescription differed from the rest of the cohort, we also carried out a within-individual analysis including these individuals only. No significant association of SSRI treatment with violent crime conviction was found for this group (HR = 0.73, 95% CI 0.45–1.17, *p =* 0.193). To account for the possibility of reverse causation, i.e., that individuals are more likely to take SSRIs after committing a crime, we excluded 996 individuals who received SSRIs within 60 d of committing a violent crime, and the risk increase remained (S2 Table). We then excluded 608 individuals who received SSRIs within 30 d of committing of a violent crime, and the risk increase remained similar (S2 Table). When we excluded those who received medication within 14 d (356 individuals) or 7 d (197 individuals) of committing a violent crime, similar risk increases were found (S2 Table). No material differences were found when we repeated this analysis with violent crime arrests as an outcome (S2 Table). When we carried out a conditional Poisson regression, a similar pattern of findings was found (incidence rate ratio for violent crime conviction = 1.18, 95% CI 1.09–1.27, *p =* 0.001). When treatment periods were defined as no breaks in prescription coverage of more than 3 or 4 mo, instead of 6 mo, no material differences were found in the within-individual models (S2 Table). When we tested for delayed treatment effects, no material differences were found for the association between SSRI treatment and violent crime convictions when the treatment period was considered to start 8 wk after the SSRI prescription was dispensed (HR = 1.21, 95% CI 1.02–1.43, *p =* 0.031). Similar effects were found when testing for SSRI discontinuation effects up to 3 wk or 12 weeks, respectively, after the last dispensed prescription (S2 Table).

## Discussion

In this study, we examined the possible association between SSRIs and violent crime using a large population-based cohort that included 856,493 individuals prescribed SSRIs. There were three main findings. First, using a within-individual design, there was an association between SSRI prescriptions and violent crime convictions. With age stratification, there was an increased hazard of violent crime convictions in individuals aged 15 to 24 y, and no significant association in older individuals. A second finding was that the association in individuals aged 15–24 y was consistent when looking at a related antidepressant (venlafaxine, a serotonin–norepinephrine reuptake inhibitor), considering four other outcomes (violent crime arrests, non-violent crime convictions and arrests, and non-fatal accidental injuries), or using another design (conditional Poisson regression). Third, the association of SSRI treatment with violent crime was not found for moderate or high SSRI use, including in those aged 15–24 y.

The finding of a modest risk association in younger people is consistent with trial data showing that children and adolescents respond differently than adults to SSRIs [34], and with reported increases in suicide-related outcomes in adolescents prescribed SSRI medication in both observational studies and clinical trials [11,32,35], although this finding is not supported by meta-analyses of trial data [36,37]. These associations may be moderated by impulsivity and risk-taking, which could explain the similar association we report with accidents, and the weaker associations with non-violent crime convictions and arrests. A recent observational investigation also found increases in suicidal behaviour in a large US cohort aged less than 25 y [38]. The US investigation found that younger people receiving the modal antidepressant dose were at increased risk of deliberate self-harm compared to adults, and this risk was further increased in individuals receiving higher doses. The apparent contrast with our findings on medication dose may be because the US study looked at risks in those initiating treatment, while our study examined all treatment periods. Importantly, this US study saw no increased hazard of self-harm in those over 25 y, analogous to our null finding for crime outcomes for those over 25 y. The reasons for the age-dependent differences are still poorly understood, but the adolescent brain may be particularly sensitive to pharmacological interference, as has been demonstrated in animal studies [38–43]. Yet, the possible adverse effects associated with SSRI use appear to be separate from its therapeutic ones; treatment effects have been demonstrated [10,37], particularly for fluoxetine and escitalopram, which are approved by the US Food and Drug Administration for treating adolescent depression [34].

The reported association between SSRIs and violent crime in young people cannot be interpreted causally because of confounding by indication. This confounding was confirmed in our study by the difference between the hazards reported in between-individual and within-individual analyses. Hence we focused on the within-individual analyses: crime outcomes in the same individuals when they were taking SSRIs compared to when they were not taking SSRIs, thus adjusting for all factors that were constant within the individual. However, this approach cannot fully account for time-varying risk factors, such as increased drug or alcohol use during periods of SSRI medication, worsening of symptoms, or a general psychosocial decline. We attempted to address the first of these by investigating substance-related convictions, one proxy for problem substance use, and recorded rates of emergency treatment for alcohol-related problems. Although we did not find an association of SSRI treatment with substance-related convictions, this was a crude outcome with an incidence of 1.7%. An alternative marker of alcohol use was the rate of emergency inpatient or outpatient treatment for alcohol intoxication or misuse, where we found some support for an increasing rate during SSRI medication, which is in keeping with one case series [44]. Although emergency treatment for alcohol intoxication or misuse is a more sensitive measure than alcohol-related crimes, it needs further clarification using prospective clinical designs. Symptom severity may moderate the association between SSRIs and the adverse outcomes reported in this study, and younger people on SSRIs may be less adherent than others, and may have more residual symptoms, such as impulsivity and hostility, which are risk factors for violence [45]. This is underscored by recent epidemiological work that suggested that depression and bipolar disorder are independent risk factors for violent crime [46,47]. If these underlying conditions are partially treated—especially in bipolar patients who are not also prescribed a mood stabiliser [27]—then residual symptoms may partly explain any association. This is further suggested by our finding that the increased hazard for violent crime conviction in younger people was not found in individuals with therapeutic SSRI exposures (≥1 DDD/day). However, the finding that there was a risk increase for non-violent crime arrests and non-violent crime convictions with SSRI use suggests a non-specificity in our findings that could be explained by time-varying confounders, or that the links may be mediated by factors that increase the risk of both violent and non-violent crime. The risk increases for non-violent crime outcomes were smaller than those for violent crime outcomes, which suggests some complexity to the possible mechanisms involved. A final possibility is that non-specific treatment factors, such as contact with health care staff, may partly explain the relationship. As most of the individuals in the sample were outpatients, and unlikely to see health care staff regularly once treatment was initiated, these factors may not be strong. In addition, the finding that violent crime conviction was inversely associated with a group of non-psychotropic medications (diuretics) suggests that, if anything, non-specific treatment effects would reduce any observed association.

Another possible challenge to the results is reverse causality—that the observed association was due to individuals taking SSRIs after being arrested for a crime (for various reasons, including coping with the anxiety and stress of arrest or that taking SSRIs might mitigate their criminal sanction). In order to address reverse causality, we excluded all persons who received SSRIs within 7, 14, 30, or 60 d after committing a violent crime, and the association between SSRI treatment and violent crime convictions remained significant, with no material change in risk. Differences between individual SSRIs were examined. The increased association between paroxetine and citalopram use and violent crime arrests could be due to their poorer efficacy and/or shorter half-life compared with other SSRIs [34,48]. Shorter half-lives are linked with withdrawal effects on discontinuation, with increased agitation and possible hostility [34,49]. Further, the single-dose and mean steady-state half-life of SSRIs with short half-lives are shorter in adolescents than in adults, and aggression thus could be a withdrawal rather than side effect [34]. In support of this, venlafaxine and, non-significantly, the heterocyclics were also linked with higher risks of violent crime convictions than SSRIs, and these medications have shorter half-lives and poorer efficacy [34,48]. Moreover, we found an increased risk of violent crime conviction for low SSRI exposure only, which is consistent with the reported links with antidepressants with shorter half-lives and case reports of increased hostility and aggression in children and adolescents at low starting doses in the first weeks of SSRI treatment [50,51]. Escitalopram, with a half-life similar to that of citalopram, was also associated with increased violent crime convictions in younger persons (S1 Table). However, any increased risks of post-cessation withdrawal for violent crime would not be included as related to SSRIs using the design in the current study if the medication was discontinued as planned, and therefore our estimates may be conservative. Our sensitivity analyses to measure post-cessation withdrawal (considering the treatment period to continue up to 3 wk or 12 wk after the date the last SSRI prescription was dispensed) nevertheless showed no material difference in the increased risk of violent crime conviction. An alternative explanation could be that the increased risk of some SSRIs is confounded by psychiatric morbidity; citalopram, escitalopram, and paroxetine are not recommended as first-line treatment for children and adolescents by the Swedish National Board of Health and Welfare, and thus are reserved for treatment-resistant patients with more severe problems [52]. Further work will need to validate differences between SSRIs and dosing strategies, and investigate underlying mechanisms in younger populations. One potentially important explanatory factor will be the timing of doses, which requires further examination.

There are two principal clinical implications arising from this study. First, no association between SSRIs and violent crime convictions was found for the majority of people who were prescribed these medications, including individuals aged 25 y and older. Second, the risk increase we report in young people is not insignificant, and hence warrants further examination. If our findings related to young people are validated in other designs, samples, and settings, warnings about an increased risk of violent behaviours while being treated with SSRIs may be needed. Any such changes to the advice given to young persons prescribed SSRIs will need to be carefully considered, as the public health benefit from decreases in violence following restrictions in SSRI use may be countered by increases in other adverse outcomes (such as more disability, rehospitalisation, or suicides) [53]. From a public health perspective, this worsening of overall morbidity and mortality might argue against restrictions on the primary care prescribing of SSRIs as long as potential risks are disclosed [54].

The present study was characterised by several strengths. The study included a large population-based cohort with longitudinal data retrieved from national registers. Information on SSRIs was complete, as each prescription that is dispensed is registered in the Swedish Prescribed Drug Register. Using a within-individual design allowed us to adjust for many unobserved factors that may bias estimates. Although a marginal structural model would have been desirable because of its ability to handle time-varying confounding factors that are also predicted by treatment history, such a model could be used only in a between-individual design that includes measures of all confounding factors. Since many confounders are most likely unobserved, we used a within-individual design as our principal approach. This allowed us to adjust for both measured and unmeasured time-invariant confounding factors, as well as for some measured time-varying confounders that are not predicted by exposure history (like sex and age). Limitations of the study include the use of diagnoses from the national patient register, which only includes diagnoses from specialists. Also, the use of official sources of data for crime outcomes is likely to underestimate true rates of crime and possibly involve selection effects. However, we tried to address such biases by using arrests with preliminary investigations in addition to convictions and also by examining accidents. It is not clear whether these findings will translate to less severe forms of violence or those not reported to the police, and triangulating the findings with information on self- or informant-reported violence will be an important future research direction. Another limitation is that detailed information about the actual prescriptions was not available. Although our data are an improvement over prescription data—as they reflect prescriptions that are dispensed by pharmacies to individuals—we were unable to account for lack of, or variations in, adherence. This problem is parallel to non-adherence in randomised controlled trials, and our within-individual estimate is comparable to the intention-to-treat analysis used in randomised controlled trials. If individuals consumed SSRIs during periods when we assumed that they were not, then this should reduce the hazards reported and would suggest that our estimates are underestimates. A possible source of underestimation is that we excluded persons who were prescribed SSRIs on only one occasion, who may have discontinued the medications due to adverse effects that were not included. We thus carried out analyses where individuals with a single prescription were included, and found no material differences in hazard of violent crime conviction. Another possible source of underestimation is that we used a conservative approach to measure the end of a treatment period (we defined this as the date the last SSRI prescription in a treatment period was dispensed), which could result in slightly lower sensitivity (i.e., individuals classified as unmedicated when truly medicated). However, sensitivity analyses using less conservative approaches to measure the end of a treatment period (3 wk and 12 wk after the last dispensed SSRI prescription in a treatment period) resulted in a similarly increased risk of violent crime conviction. Sweden has prescription rates of SSRIs that are higher than the average for Europe (5-y mean DDD/1,000 individuals/day: Sweden = 70.1; across 29 European countries = 40.0) [14] and similar to the US (10.8% treated in our cohort between 2006–2009 compared to 10.1% treated in the US in 2005) [55]. In relation to criminality, Sweden has similar police-reported assault rates as the US [56]. Finally, there might be residual confounding for the within-individual estimates due to unmeasured time-varying confounders. However, we are not aware of any statistical method that also allows adjustment for unmeasured time-varying confounders.

In summary, we demonstrated associations between SSRIs and violent crime that vary by age group. The clinical and public health implications of this require careful consideration, and validation in other settings.

## Supporting Information

S1 STROBE STROBE checklist. (DOCX)

S1 Methods Definitions of SSRIs and other psychotropic medications, crimes, and hospitalisations. (DOCX)

S1 Table Violent crime convictions and alternative outcomes in individuals treated with SSRI medication compared to non-treatment periods in the same person, stratified by age using stratified Cox regression models. (DOCX)

S2 Table Violent crime convictions and arrests in individuals treated with SSRI medication compared to non-treatment periods in the same person using conditional Poisson regression, stratified Cox regression models for alternative treatment periods, and analyses excluding individuals who received SSRIs after committing a crime. (DOCX)

## Abbreviations

DDD: defined daily dose
HR: hazard ratio
SSRI: selective serotonin reuptake inhibitor

## References

[1] Pratt LA, Brody DJ, Gu Q. Antidepressant use in persons aged 12 and over: United States, 2005–2008. NCHS data brief, no 76. Hyattsville (Maryland): National Center for Health Statistics; 2011. 22617183

[2] Berkrot B. Global drug sales to top $1 trillion Thomson Reuters 20 4 2010 Available: http://www.reuters.com/article/idUKTRE63J0Y520100420. Accessed 31 March 2015.

[3] Aguglia E, Ravasio R, Simonetti M, Pecchioli S, Mazzoleni F. Use and treatment modalities for SSRI and SNRI antidepressants in Italy during the period 2003–2009. Curr Med Res Opin. 2012;28:1475–1484. 22809113 10.1185/03007995.2012.713341

[4] Lockhart P, Guthrie B. Trends in primary care antidepressant prescribing 1995–2007: a longitudinal population database analysis. Br J Gen Pract. 2011;61:e565–e572. 10.3399/bjgp11X593848 22152736 PMC3162179

[5] Skaer TL, Sclar DA, Robison LM. Trends in prescriptions for antidepressant pharmacotherapy among US children and adolescents diagnosed with depression, 1990 through 2001: an assessment of accordance with treatment recommendations from the American Academy of Child and Adolescent Psychiatry. Clin Ther. 2009;31:1478–1487. 10.1016/j.clinthera.2009.07.002 19698905

[6] D’Souza P, Jago C. Spotlight on depression: a pharma matters report. Drugs Today (Barc). 2014;50:251–67. 24696870 10.1358/dot.2014.50.3.2134450

[7] Stone M, Laughren T, Jones ML. Risk of suicidality in clinical trials of antidepressants in adults: analysis of proprietary data submitted to US Food and Drug Administration. BMJ 2009;339:b2880 10.1136/bmj.b2880 19671933 PMC2725270

[8] Masi G, Liboni F, Brovedani P. Pharmacotherapy of major depressive disorder in adolescents. Exp Opin Pharmacother. 2010;11:375–386. 10.1517/14656560903527226 20102303

[9] Adegbite-Adeniyi C, Gron B, Rowles BM, Demeter CA, Findling RL. An update on antidepressant use and suicidality in pediatric depression. Exp Opin Pharmacother. 2012;13:2119–2130. 10.1517/14656566.2012.726613 22984934

[10] Hetrick SE, McKenzie JE, Cox GR, Simmons MB, Merry SN. Newer generation antidepressants for depressive disorders in children and adolescents. Cochrane Database Syst Rev. 2012;11:CD004851 10.1002/14651858.CD004851.pub3 23152227 PMC8786271

[11] Barbui C, Esposito E, Cipriani A. Selective serotonin reuptake inhibitors and risk of suicide: a systematic review of observational studies. CMAJ. 2009;180:291–297. 10.1503/cmaj.081514 19188627 PMC2630355

[12] Gibbons RD, Brown CH, Hur K, Marcus SM, Bhaumik DK, Erkens JA, et al Early evidence on the effects of regulators’ suicidality warnings on SSRI prescriptions and suicide in children and adolescents. Am J Psychiatry. 2007;164:1356–1363. 17728420 10.1176/appi.ajp.2007.07030454

[13] Hall WD, Lucke J. How have the selective serotonin reuptake inhibitor antidepressants affected suicide mortality? Aust N Z J Psychiatry. 2006;40:941–950. 17054562 10.1080/j.1440-1614.2006.01917.x

[14] Olfson M, Shaffer D, Marcus SC, Greenberg T. Relationship between antidepressant medication treatment and suicide in adolescents. Arch Gen Psychiatry. 2003;60:978–982. 14557142 10.1001/archpsyc.60.9.978

[15] Gusmao R, Quintao S, McDaid D, Arensman E, van Audenhove C, Coffey C, et al Antidepressant utilization and suicide in Europe: an ecological multi-national study. PLoS ONE. 2013;8:e66455 23840475 10.1371/journal.pone.0066455 PMC3686718

[16] Healy D, Herxheimer A, Menkes DB. Antidepressants and violence: problems at the interface of medicine and law. PLoS Med. 2006;3:e372 16968128 10.1371/journal.pmed.0030372 PMC1564177

[17] Marcotte DE, Markowitz S. A cure for crime? Psycho-pharmaceuticals and crime trends. J Policy Anal Manag. 2011;30:29–56. 10.1002/pam.20544 21465827

[18] Bouvy PF, Liem M. Antidepressants and lethal violence in the Netherlands 1994–2008. Psychopharmacol. 2012;222:499–506. 10.1007/s00213-012-2668-2 PMC3395354 22395429

[19] Moore TJ, Glenmullen J, Furberg CD. Prescription drugs associated with reports of violence towards others. PLoS ONE. 2010;5:e15337 10.1371/journal.pone.0015337 21179515 PMC3002271

[20] Rouve N Bagheri H, Telmon N, Pathak A, Franchitto N, Schmitt L, et al Prescribed drugs and violence: a case/noncase study in the French PharmacoVigilance Database. Eur J Clin Pharmacol. 2011;67:1189–1198. 10.1007/s00228-011-1067-7 21655992

[21] Madsen IE, Burr H, Diderichsen F, Pejtersen JH, Borritz M, Bjorner JB, et al Work-related violence and incident use of psychotropics. Am J Epidemiol. 2011;174:1354–1362. 10.1093/aje/kwr259 22038105

[22] Bramness JG, Walby F. Ecological studies and the big puzzle of failing suicide rates. Acta Psychiat Scand. 2009;119:169–170. 10.1111/j.1600-0447.2008.01318.x 19178393

[23] Gibbons RD, Mann JJ. Strategies for quantifying the relationship between medications and suicidal behaviour. What has been learned? Drug Saf. 2011;34:375–395. 10.2165/11589350-000000000-00000 21513361

[24] Lichtenstein P, Halldner L, Zetterqvist J, Sjölander A, Serlachius E, Fazel S, et al Medication for attention deficit–hyperactivity disorder and criminality. N Engl J Med. 2012;367:2006–2014. 10.1056/NEJMoa1203241 23171097 PMC3664186

[25] Chang Z, Lichtenstein P, D’Onofrio BM, Sjölander A, Larsson H. Serious transport accidents in adults with attention-deficit/hyperactivity disorder and the effect of medication: a population-based study. JAMA Psychiatry. 2014;71:319–325. 10.1001/jamapsychiatry.2013.4174 24477798 PMC3949159

[26] Chen Q, Sjolander A, Runeson B, D’Onofrio BM, Lichtenstein P, Larsson H. Drug treatment for attention-deficit/hyperactivity disorder and suicidal behaviour: register based study. BMJ. 2014;348:g3769 10.1136/bmj.g3769 24942388 PMC4062356

[27] Fazel S, Zetterqvist J, Larsson H, Långström N, Lichtenstein N. Antipsychotics, mood stabilisers, and risk of violent crime. Lancet. 2014;384:1206–1214. 10.1016/S0140-6736(14)60379-2 24816046 PMC4165625

[28] Wettermark B, Hammar N, Fored CM, Leimanis A, Otterblad Olausson P, Bergman U, et al The new Swedish Prescribed Drug Register—opportunities for pharmacoepidemiological research and experience from the first six months. Pharmacoepidemol Drug Saf. 2007;16:726–735. 10.1002/pds.1294 16897791

[29] Isacsson G, Ahlner J. Antidepressants and the risk of suicide in young persons—prescription trends and toxicological analyses. Acta Psychiatr Scand. 2014;129:296–302. 10.1111/acps.12160 23773187 PMC4224008

[30] Fazel S, Långström N, Hjern A, Grann M, Lichtenstein P. Schizophrenia, substance abuse, and violent crime. JAMA 2009;301:2016–2023. 10.1001/jama.2009.675 19454640 PMC4905518

[31] Allison PD. Fixed-effects partial likelihood for repeated events. Sociol Methods Res. 1996;25:207–222.

[32] Jick H, Kaye JA, Jick SS. Antidepressants and the risk of suicidal behaviours. JAMA. 2004;292:338–343. 15265848 10.1001/jama.292.3.338

[33] WHO Collaborating Center for Drug Statistics Methodology. ATC/DDD index 2015. Available: http://www.whocc.no/atc_ddd_index/. Accessed 31 March 2015.

[34] Sakolsky D, Birmaher B. Developmentally informed pharmacotherapy for child and adolescent depressive disorders. Child Adolesc Psychiatr Clin N Am. 2012;21:313–325. 10.1016/j.chc.2012.01.005 22537729

[35] Hammad TA, Laughren T, Racoosin J. Suicidality in pediatric patients treated with antidepressant drugs. Arch Gen Psychiatry. 2006;63:332–339. 16520440 10.1001/archpsyc.63.3.332

[36] Carpenter DJ, Fong R, Kraus JE, Davies JT, Moore C, Thase ME. Meta-analysis of efficacy and treatment-emergent suicidality in adults by psychiatric indication and age subgroup following initiation of paroxetine therapy: a complete set of randomized placebo-controlled trials. J Clin Psychiatry. 2011;72:1503–1514. 10.4088/JCP.08m04927blu 21367354

[37] Gibbons RD, Brown CH, Hur K, Davis JM, Mann JJ. Suicidal thoughts and behaviour with antidepressant treatment reanalysis of the randomized placebo-controlled studies of fluoxetine and venlafaxine. JAMA Psychiatry. 2012;69:580–587. 10.1001/archgenpsychiatry.2011.2048 PMC3367101 22309973

[38] MillerM, SwansonSA, AzraelD, PateV, StürmerT. Antidepressant dose, age, and the risk of deliberate self-harm. JAMA Intern Med. 2014;174:899–909. 10.1001/jamainternmed.2014.1053 24782035

[39] Bylund DB, Reed AL. Childhood and adolescent depression: why do children and adults respond differently to antidepressant drugs? Neurochem Int. 2007;51:246–253. 17664028 10.1016/j.neuint.2007.06.025 PMC2694752

[40] Karanges E, McGregor IS. Antidepressants and adolescent brain development. Future Neurol. 2011;6:783–808.

[41] Klomp A, Tremoleda JL, Wylezinska M, Nederveen AJ, Feenstra M, Gsell W, et al Lasting effects of chronic fluoxetine treatment on the late developing rat brain: age-dependent changes in the serotonergic neurotransmitter system assessed by pharmacological MRI. Neuroimage. 2012;59:218–226. 10.1016/j.neuroimage.2011.07.082 21840402

[42] Bouet V, Klomp A, Freret T, Wylezinska-Arridge M, Lopez-Tremoleda J, Dauphin F, et al Age-dependent effects of chronic fluoxetine treatment on the serotonergic system one week following treatment. Psychopharmacology (Berl). 2012;221:329–339. 22205158 10.1007/s00213-011-2580-1

[43] Klomp A, Václavu L, Meerhoff GF, Reneman L, Lucassen PJ. Effects of chronic fluoxetine treatment on neurogenesis and tryptophan hydroxylase expression in adolescent and adult rats. PLoS ONE. 2014;9:e97603 10.1371/journal.pone.0097603 24827731 PMC4020860

[44] Menkes DB, Herxheimer A. Interaction between antidepressants and alcohol: signal amplification by multiple case reports. Int J Risk Saf Med. 2014 26:163–170. 10.3233/JRS-140632 25214162

[45] Loeber R, Menting B, Lynam DR. Findings from the Pittsburgh Youth Study: cognitive impulsivity and intelligence as predictors of the age–crime curve. J Am Acad Child Adolesc Psychiatry. 2012;51:1136–1149. 10.1016/j.jaac.2012.08.019 23101740

[46] Webb RT, Lichtenstein P, Larsson H, Geddes JR, Fazel S. Suicide, hospital presenting suicide attempts, and criminality in bipolar disorder: examination of risk for multiple adverse outcomes. J Clin Psychiatry. 2014;75:e809–e816. 10.4088/JCP.13m08899 25191918 PMC4226039

[47] Fazel S, Wolf A, Chang Z, Larsson H, Goodwin GM, Lichtenstein P. Depression and violence: a Swedish population study. Lancet Psychiatry. 2015;2:224–232. 26236648 10.1016/S2215-0366(14)00128-X PMC4520382

[48] Cipriani A, Furukawa TA, Salanti G, Geddes JR, Higgins JP, Churchill R, et al Comparative efficacy and acceptability of 12 new-generation antidepressants: a multiple-treatments meta-analysis. Lancet. 2009;373:746–758. 10.1016/S0140-6736(09)60046-5 19185342

[49] Renoir T. Selective serotonin reuptake inhibitor antidepressant treatment discontinuation syndrome: a review of the clinical evidence and the possible mechanisms involved. Front Pharmacol. 2013;16:4–45. 10.3389/fphar.2013.00045 PMC3627130 23596418

[50] Cheung AH, Dewa CS, Levitt AJ. Clinical review of mania, hostility and suicide-related events in children and adolescents treated with antidepressants. Paediatr Child Health. 2005;10:457–463. 19668657 PMC2722596

[51] Kratochvil CJ, Vitiello B, Walkup J, Emslie G, Waslick BD, Weller EB, et al Selective serotonin reuptake inhibitors in pediatric depression: is the balance between benefits and risks favorable? J Child Adolesc Psychopharmacol. 2006;16:11–24. 16553525 10.1089/cap.2006.16.11

[52] National Board of Health and Welfare. [National guidelines for care in depression and anxiety disorders] Västerås: National Board of Health and Welfare; 2010.

[53] Lu CY, Zhang F, Lakoma MD, Madden JM, Rusinak D, Penfold RD, et al Changes in antidepressant use by young people and suicidal behaviour after FDA warnings and media coverage: quasi-experimental study. BMJ 2014;348:g3596 10.1136/bmj.g3596 24942789 PMC4062705

[54] Busch SH, Barry CL. Pediatric antidepressant use after the black-box warning. Health Aff (Millwood). 2009;28:724–733. 19414881 10.1377/hlthaff.28.3.724 PMC2768536

[55] Olfson M, Marcus SC. National patterns in antidepressant medication treatment. Arch Gen Psychiatry. 2009;66:848–856. 10.1001/archgenpsychiatry.2009.81 19652124

[56] Farrington D, Langan P, Tonrym M, editors. Cross-national studies in crime and justice Washington (District of Columbia): Bureau of Justice Statistics; 2004.

