# Supplementary material for: Selective Serotonin Reuptake Inhibitors and Violent Crime: A Cohort Study
Source: PLoS Med. 2015 Sep 15;12(9):e1001875. doi: 10.1371/journal.pmed.1001875 (PMC4570770; doi:10.1371/journal.pmed.1001875)
Supplement: S1 STROBE — (DOCX) [file pmed.1001875.s001.docx]

STROBE Statement—checklist of items that should be included in reports of observational studies

|  | Item No | Recommendation |
| --- | --- | --- |
| **Title and abstract** | 1 | (*a*) Indicate the study’s design with a commonly used term in the title or the abstract  **Within the Title on page 1:**  **“Selective serotonin reuptake inhibitors and violent crime: a cohort study”** |
|  |  | (*b*) Provide in the abstract an informative and balanced summary of what was done and what was found  **In the Methods and Findings sections of the Abstract on page 2 we wrote:**  **“Methods: Using Swedish national registers, we extracted information on 856,493 individuals who were prescribed SSRIs, and subsequent violent crimes during 2006 through 2009. We used stratified Cox regression analyses to compare the rate of violent crime while individuals were prescribed these medications with the rate in the same individuals while not receiving medication. Adjustments were made for other psychotropic medications. Information on all medications was extracted from the Prescribed Drug Register, with complete national data on all collected medications. Convicted violent crimes were extracted from the National Crime Register. Findings: Using within-individual association, there was an overall association between SSRIs and violent convictions (HR=1.19, 95% CI: 1.08-1.32, p-value =0.0005). On age stratification, there was a significant association between SSRIs and violent convictions for individuals aged 15 to 24 (HR=1.43, 1.19-1.73, p=0.0002). However, there were no significant associations in those aged 25-34 (HR=1.20, 0.95-1.52, p=0.1252), in those aged 35-44 (HR=1.06, 0.83-1.35, p=0.6662) or in those older than 45 (HR=1.07, 0.84-1.35, p=0.1403). Associations in those aged 15 to 24 were also found for violent arrests (HR=1.28, 1.16-1.41, p=0.0001), non-violent convictions (HR=1.22, 1.10-1.34, p=0.0001) and arrests (HR=1.13, 1.07-1.20, p=0.0001), and non-fatal hospitalized accidents (HR=1.29, 1.22-1.36, p=0.0001)”.** |
| Introduction | | |
| Background/rationale | 2 | Explain the scientific background and rationale for the investigation being reported  **In the Introduction on page 3 we wrote:**  **“Selective serotonin reuptake inhibitors (SSRIs) are among the most widely prescribed psychiatric medications in many countries [1-6]. At the same time, concerns about their adverse effects, including suicide and violence, have been widely discussed and remain controversial. Observational and trial data have shown that, although SSRIs appear not elevate the risk for suicidal behaviour in adults, they may increase the risk of suicide ideation in children, adolescents, and young adults. This age-related association is weak and consistent across studies [7-11] but inconsistently supported by ecological data [12-16].”** |
| Objectives | 3 | State specific objectives, including any prespecified hypotheses  **In the Introduction on pages 3 and 4 we wrote:**  **“Our objective was thus to investigate the association between SSRIs and violence outcomes by linking data from Swedish national registers on individual SSRIs prescriptions, use of other psychotropic drugs, and violent crimes in a large population-based cohort. We have primarily used a ‘within-individual’ design [25-28], where the risk of violent crime is determined when an individual is taking an SSRI as compared to when the same person is not. Using this design, all time-invariant factors (i.e. genetic factors, all factors before the start of follow-up, and factors that remain constant during follow-up) are accounted for, thus more fully adjusting for unmeasured time-invariant confounding and confounding by indication than other observational designs, but not accounting for time-varying factors such as symptom severity. We hypothesized that any associations between SSRI medication and violent outcomes would not be demonstrated using a within-individual design, including in different age groups”.** |
| Methods | | |
| Study design | 4 | **Present key elements of study design early in the paper**  **In the Methods on page 5 we wrote:**  **“In the total population of Sweden aged 15 and older in 2006 (n=7,917,854), and residing in Sweden during follow-up (January 1, 2006 to December 31, 2009), we identified 856,493 individuals who were prescribed SSRI treatment. Information on individuals receiving SSRI treatment was collected from Swedish population-based registers with national coverage, and registers were linked using each individual’s unique identification number”.** |
| Setting | 5 | Describe the setting, locations, and relevant dates, including periods of recruitment, exposure, follow-up, and data collection  **In the Methods on page 5 we wrote:**  **“In the total population of Sweden aged 15 and older in 2006 (n=7,917,854), and residing in Sweden during follow-up (January 1, 2006 to December 31, 2009), we identified 856,493 individuals who were prescribed SSRI treatment”.**  **Furthermore we wrote in the Methods on page 5:**  **“Information on medication and date of the collected prescription was extracted from the Prescribed Drug Register, with complete national data on all prescribed and collected medical drugs from all pharmacies in Sweden since July 2005”.**  **In the Methods on page 6 we wrote:**  **“Information on convictions for violent crimes for individuals aged 15 and older (the age of criminal responsibility) was extracted from the National Crime Register. Violent crimes were defined as crimes against person as per previous work [31] (see appendix methods for definitions).”** |
| Participants | 6 | (*a*) *Cohort study*—Give the eligibility criteria, and the sources and methods of selection of participants. Describe methods of follow-up  **In the Methods on page 5 we wrote:**  **In our initial analysis, we included all individuals with collected SSRI prescriptions. However, as we wanted to restrict the sample to those adherent with SSRIs, individuals with a single SSRI prescription within a six-month-period were excluded from all further analyses as no assumptions could be made about their medication adherence. A separate analysis was also carried out including only individuals with a single collected prescription. A treatment period was thus defined as a series of SSRI prescriptions with no more than six months between two consecutive prescriptions. The start of a treatment period was defined as the first date of an SSRI prescription during our follow-up. The end of a treatment period was defined as the date of the last SSRI prescription in that treatment period. Periods of more than six months between prescriptions were considered as non-treatment periods. A new treatment period was considered to have started at the first date of the next series of consecutive prescriptions (see appendix for details on SSRI medications). For individuals with a single prescription, the start their treatment period was defined as the first date of their collected prescription, and the end of that treatment period was defined as 14 days after the collected prescription.**  **In the Methods on page 6 we wrote:**  **“Information on convictions for violent crimes for individuals aged 15 and older (the age of criminal responsibility) was extracted from the National Crime Register. Violent crimes were defined as crimes against person as per previous work [31] (see appendix methods for definitions).**  **Examinations of individual SSRIs and alternative outcomes were also carried out, including (1) convictions for substance-related crimes; (2) convictions for non-violent crimes, (3) arrests, as distinct to convictions, for violent crimes; (4) arrests for substance-related crimes; (5) arrests for non-violent crimes; (6) non-fatal injuries from accidents; (7) alcohol intoxications; (8) and psychiatric hospitalisations (see appendix methods for details on alternative outcomes).”**  *Case-control study*—Give the eligibility criteria, and the sources and methods of case ascertainment and control selection. Give the rationale for the choice of cases and controls  *Cross-sectional study*—Give the eligibility criteria, and the sources and methods of selection of participants |
|  |  | (*b*) *Cohort study*—For matched studies, give matching criteria and number of exposed and unexposed  **N/A**  *Case-control study*—For matched studies, give matching criteria and the number of controls per case |
| Variables | 7 | Clearly define all outcomes, exposures, predictors, potential confounders, and effect modifiers. Give diagnostic criteria, if applicable  **In the Methods on pages 5-6 we define all exposures, outcomes, and potential confounders:**  **“SSRI treatment**  **Information on medication and date of the collected prescription was extracted from the Prescribed Drug Register, with complete national data on all prescribed and collected medical drugs from all pharmacies in Sweden since July 2005 [29]. A previous comparison between post-mortem toxicology and SSRI purchases in the Prescribed Drug Register indicated good medication compliance [30].**  **In our initial analysis, we included all individuals with collected SSRI prescriptions. However, as we wanted to restrict the sample to those adherent with SSRIs, individuals with a single SSRI prescription within a six-month-period were excluded from all further analyses as no assumptions could be made about their medication adherence. A separate analysis was also carried out including only individuals with a single collected prescription. A treatment period was thus defined as a series of SSRI prescriptions with no more than six months between two consecutive prescriptions. The start of a treatment period was defined as the first date of an SSRI prescription during our follow-up. The end of a treatment period was defined as the date of the last SSRI prescription in that treatment period. Periods of more than six months between prescriptions were considered as non-treatment periods. A new treatment period was considered to have started at the first date of the next series of consecutive prescriptions (see appendix for details on SSRI medications). For individuals with a single prescription, the start their treatment period was defined as the first date of their collected prescription, and the end of that treatment period was defined as 14 days after the collected prescription.**  **Other psychotropic medications**  **Adjustments were made for concurrent psychotropic medications other than SSRIs (see appendix for details). Treatment periods were defined in the same manner as SSRI treatment periods.**  **Violent crimes**  **Information on convictions for violent crimes for individuals aged 15 and older (the age of criminal responsibility) was extracted from the National Crime Register. Violent crimes were defined as crimes against person as per previous work [31] (see appendix methods for definitions).**  **Alternative outcomes**  **Examinations of individual SSRIs and alternative outcomes were also carried out, including (1) convictions for substance-related crimes; (2) convictions for non-violent crimes, (3) arrests, as distinct to convictions, for violent crimes; (4) arrests for substance-related crimes; (5) arrests for non-violent crimes; (6) non-fatal injuries from accidents; (7) alcohol intoxications; (8) and psychiatric hospitalisations (see appendix methods for details on alternative outcomes)”.**  **In the Appendix Methods on pages 45-48 we provide additional information on these measures:**  **“SSRI medications**  **Among the 856,493 individuals who were prescribed SSRI treatment, 65,862 individuals were prescribed fluoxetine (N06AB03), 389,857 citalopram (N06AB04), 46,615 paroxetine (N06AB05), 215,873 sertraline (N06AB06), 1198 fluvoxamine (N06AB08), and 84,934 individuals were prescribed escitalopram (N06AB10).**  **Crimes**  **Information on convictions for violent crimes included attempted, completed and aggravated forms of: homicide, manslaughter, unlawful threats, harassment, robbery, arson, assault, assault on an official, kidnapping, stalking, coercion, and all sexual offences, and the date of perpetration was identified for each conviction. Convictions for substance-related crimes included manufacturing alcohol, driving under the influence of alcohol or illicit substances, smuggling illicit substances, manufacturing illicit substances, supplying illicit substances, possession of illicit substances, and personal use of illicit substances. Convictions for non-violent crimes included all offences other than violent crimes and substance-related crimes. Convictions were extracted from the National Crime Register, including all convictions in Swedish district courts [57].**  **Conviction data were used because individuals are convicted as guilty regardless of mental illness in Sweden, thus conviction data included persons who received custodial or noncustodial sentences and individuals transferred to forensic hospital (e.g., individuals who were deemed to have suffered from severe mental disorder at the time of the offense). Furthermore, conviction data also included those cases in which the prosecutor decided to caution or fine (e.g., less serious sexual crimes and some juvenile cases). In addition, though sentencing decisions may vary according to background factors, plea-bargaining at the conviction stage is not part of the Swedish legal system. Therefore, conviction data more accurately reflect the extent of officially resolved criminality in the population. The crime register has total national coverage—only 0.05% of all registered convictions had incomplete personal identification numbers in another study [58].**  **Arrests, as distinct to convictions, were identified in the Register of Persons Suspected of Offenses, which includes all individuals suspected of crime after a completed investigation by police, the customs authority, or the prosecution service [57], regardless of whether suspicion leads to prosecution or conviction.**  **Other psychotropic medications**  **Adjustments were made for concurrent psychotropic medications other than SSRIs, and included: Antipsychotics (N05A, except N05AN01), hypnotics, sedatives and anxiolytics (N05B and N05C), drugs used in addictive disorders (N07B), mood stabilizers (N03AF01, N03AF02, N03AG01, N05AN01, N03AX09), antiepileptics (N03A, except mood stabilizers), and antidepressant medications other than SSRIs (venlafaxine [N06AX16], duloxetine [N06AX21], tricyclics [N06AA04, N06AA09, N06AA10], heterocyclics [N06AA21, N06AX03], mirtazapine [N06AZ11], monoamine oxidase inhibitors, non-selective [N06AF], moclobemide [N06AG02], and bupropion [N06AX12]. Antidepressant medications other than SSRIs were also used as alternative exposure in sensitivity analyses. Information on medication and date of the collected prescription was extracted from the Prescribed Drug Register [29].**  **Non-psychotropic medications**  **In further sensitivity analyses, diuretics (C03) were used as an alternative exposure. Information was collected from the Prescribed Drug Register [29].**  **Psychiatric diagnoses**  **Psychiatric diagnoses were defined as: psychotic disorders (ICD-8: 295, 297-299, ICD-9: 295, 297, 298, ICD-10: F20-F29), mood disorders (ICD-8: 296, 300.4, ICD-9: 296, 300E, 311, ICD-10: F30-F39), anxiety, dissociative, stress-related, and somatoform disorders (ICD-8: 300 except 300.4, 307, ICD-9: 300 except 300.E, 308-309, ICD-10: F40-F45, F48, 6), eating disorders (ICD-9: 307B, 307F, ICD-10: F50), and psychoactive substance misuse (ICD-8: 291, 303, 304, ICD-9: 291, 303, 304, 305A, 305X, ICD-10: F10-F19). Diagnoses were collected from the patient register [59], which includes diagnoses from both hospitalisations and outpatient visits in specialised care.**  **Non-fatal injuries from accidents**  **These were defined as emergency inpatient or outpatient episodes of: transport accidents (ICD10: V01-V99), other external causes of accidental injury (ICD10: W00-W99, X00-X49, X58-X59), sequelae of external causes of morbidity and mortality (ICD10: Y85, Y86), supplementary factors related to morbidity and mortality classified elsewhere (ICD10: Y90-Y98), and injury and certain other consequences of external causes, excluding poisoning (ICD10: S00-S99, T00-T35, T90-T94). Diagnoses were collected from the patient register [59]. Diagnoses received during planned visits (i.e. follow-ups and referrals) were excluded from the analyses. Although this is a more conservative estimate, this measure was used to avoid overestimation of diagnoses, as the diagnosis that is the reason for treatment initiation is also coded during follow-ups and referrals.**  **Alcohol intoxications**  **Alcohol intoxications were defined as emergency inpatient or outpatient episodes of mental and behavioural disorders due to the use of alcohol (ICD10: F10), toxic effect of alcohol (ICD10: T51), and supplementary factors; evidence of alcohol involvement determined by blood alcohol level (ICD10: Y90) or by level of intoxication (ICD10: Y91). Diagnoses were collected from the patient register [59], and diagnoses received during planned visits (i.e. follow-ups and referrals) were excluded from the analyses.**  **Psychiatric hospitalisations**  **Information on emergency inpatient or outpatient episodes in psychiatric care was extracted from the patient register [59], and included care in general psychiatry, child and adolescent psychiatry, geriatric psychiatry and forensic psychiatry. Planned visits (i.e. follow-ups and referrals) in psychiatric care were excluded. Because of the risk of reverse causation between SSRIs and psychiatric hospitalisations (i.e. being treated at a psychiatric hospital increases the probability of subsequent SSRI treatment), the first eight first weeks of SSRI treatment were removed when analysing of this outcome”.** |
| Data sources/ measurement | 8* | For each variable of interest, give sources of data and details of methods of assessment (measurement). Describe comparability of assessment methods if there is more than one group  **In the Method on page 5 we wrote:**  **“Information on individuals receiving SSRI treatment was collected from Swedish population-based registers with national coverage, and registers were linked using each individual’s unique identification number”.**  **Furthermore:**  **Information on individuals in Sweden aged 15 and older and residing in Sweden at the start of follow-up was provided by Statistics Sweden. This included information on age and sex.**  **Information on all medications was collected from the Prescribed Drug Register.**  **Information on convictions of crimes was extracted from the National Crime Register.**  **Information on arrests was identified in the Register of Persons Suspected of Offenses.**  **Information on psychiatric diagnoses, psychiatric hospitalisations, non-fatal accidents and alcohol intoxication was extracted from the Patient Register.**  **Follow-up time was adjusted for migration, periods in prison or institutional youth care, hospitalisation, and death through linkage to the Migration Register, Prison Register, Patient Register and Cause of Death Register.** |
| Bias | 9 | Describe any efforts to address potential sources of bias  **Our choice of principal analytic strategy was based on reducing bias. In the** **Introduction on page 4 we wrote:**  **“Using this design, all time-invariant factors (i.e. genetic factors, all factors before the start of follow-up, and factors that remain constant during follow-up) are accounted for, thus more fully adjusting for unmeasured time-invariant confounding and confounding by indication than other observational designs, but not accounting for time-varying factors such as symptom severity”.**  **Furthermore, additional efforts to address potential bias were made. In the Sensitivity Analyses on pages 8-10 we wrote:**  **In sensitivity analyses, within-individual stratified Cox proportional hazards regressions were carried with the following alternative outcomes; convictions for non-violent crimes, convictions for substance-related crimes, arrests for violent crimes, arrests for non-violent crimes, arrests for substance-related crimes, non-fatal injuries from accidents, alcohol intoxications, and psychiatric hospitalisations. Furthermore, each SSRI medication was analysed separately, and periods of using of two or more SSRI medications were excluded to adjust for switching effects between SSRI medications. Furthermore, all SSRIs were entered in the same model as covariates to adjust for concurrent use of other SSRIs. Analyses were also stratified on type of SSRI medication with suspicions of violent crimes as an alternative outcome. Additionally, other antidepressants (venlafaxine, duloxetine, tricyclics, heterocyclics, mirtazapine, moclobemide, and bupropion) were used as an alternative exposure for convicted violent crimes. Treatment periods for these antidepressants were defined in the same manner as SSRI treatment periods. Finally, diuretics were used as an alternative exposure for convicted violent crimes to test the model.**  **For individuals who started SSRI treatment after being convicted of a violent crime, the number of days between the date of committing the crime and the start of SSRI treatment was calculated. To exclude the possibility of reverse causation, i.e. if committing a violent crime increased the probability of subsequent SSRI treatment, new within-individual stratified Cox proportional hazards regressions were carried out excluding all individuals who received SSRI treatment within 7, 14, 30 or 60 days, respectively, after committing a violent crime from these analysis.**  **Finally, the robustness of results was tested by undertaking four alternative analyses: First, a conditional Poisson regression examined how changes in medication exposure were associated with changes in violent crimes within the same person, thus adjusting for time-invariant confounders. Second, we repeated the main models with different treatment periods; (a) a treatment period was defined as a series of SSRI prescriptions with no more than three months between two consecutive prescriptions, and (b) with no more than four months between two consecutive prescriptions. Third, we tested for delayed onsets of action of SSRIs by setting the first day of medication to eight weeks after the first collected prescription starting each medication period. Fourth, we tested for SSRI discontinuation effects by extending the end of a treatment period to; (a) three weeks after the date of the last SSRI prescription in that treatment period, and (b) twelve weeks after the date of the last SSRI prescription in that treatment period, respectively.** |
| Study size | 10 | Explain how the study size was arrived at  **We identified all individuals aged 15 and older (i.e. the age of criminal responsibility) and residing in Sweden at the start of follow-up in 2006** |
| Quantitative variables | 11 | Explain how quantitative variables were handled in the analyses. If applicable, describe which groupings were chosen and why  **In the Statistical Analyses on pages 6-8 we wrote:**  **“Individuals were followed from January 1, 2006 to December 31, 2009, and follow-up was adjusted for migration, periods in prison or institutional youth care, hospitalisation, and death through linkage to the Migration Register, Prison Register, Patient Register and Cause of Death Register. Unobservable time, i.e. time abroad, in prison, and in hospital, was removed (truncated) from the follow-up time. Time after hospital discharge, release from prison, or immigration, was added to the observable cohort again.**  **A between-individual Cox proportional hazards regression compared average rates of violent crime convictions during SSRI medication with rates during non-medication for all individuals. In this analysis, follow-up period was split up into the period before the first outcome, periods between outcomes, and the period after the last outcome. Time at risk was measured from the start of each period, and medication was used as a time-varying covariate. Robust standard errors were calculated to account for correlations between periods within the same individual. This analysis was adjusted for sex and age.**  **The principal analyses were within-individual stratified Cox proportional hazards regressions, with each individual entering as a separate stratum in the analysis and serving as his/her own control. The obtained hazard ratio is thus adjusted for (i.e. stratified on) all potential time-invariant confounders within each individual. To adjust for age, which is a time-varying potential confounder, age was added to the model as a time-varying covariate, with one factor for each whole year. In the within-individual stratified Cox proportional hazards regression, only individuals who change medication status contribute directly to the estimate. All other individuals contribute indirectly through the estimates of other covariates. Since the covariates in the within-individual stratified Cox proportional hazards regression are time-varying, we did not test for the proportional hazards assumption. More information on this approach is provided here [32], and has been applied in studies of ADHD medication, antipsychotics and mood stabilisers [25-28]. To ensure that outcomes were measured appropriately, all crimes were included from the date of perpetration (rather than conviction), and those with uncertain date of perpetration were excluded from the analyses, resulting in the exclusion of 1.3% (1241) violent convictions, 1.0% (9108) non-violent convictions, and 1.8% (5187) substance-related convictions during 2006 to 2009.**  **To test for confounding by other psychotropic medications, we first adjusted for concurrent exposure to other psychotropic medications as a time-varying covariate. Second, we excluded individuals with other psychotropic medications during follow-up from the within-individual stratified Cox proportional hazards regression. Furthermore, analyses were stratified on sex, on age, and on type of SSRI medication (fluoxetine, citalopram, paroxetine, sertraline, and escitalopram, respectively).**  **To estimate cumulative exposure of SSRIs, the defined daily dose (DDD) of SSRI medication [30] was calculated through summing collected medication, and then dividing the sum by the number of days in the treatment period. DDDs were categorized into 4 groups; (1) no exposure; (2) low SSRI exposure (<1 DDD/day); (3) moderate SSRI exposure (1-2 DDD/day), and; (4) high SSRI exposure (>2 DDD/day)”.** |
| Statistical methods | 12 | (*a*) Describe all statistical methods, including those used to control for confounding  **In the Statistical Analyses on pages 6-7 we wrote:**  **“A between-individual Cox proportional hazards regression compared average rates of violent crime convictions during SSRI medication with rates during non-medication for all individuals. In this analysis, follow-up period was split up into the period before the first outcome, periods between outcomes, and the period after the last outcome. Time at risk was measured from the start of each period, and medication was used as a time-varying covariate. Robust standard errors were calculated to account for correlations between periods within the same individual. This analysis was adjusted for sex and age.**  **The principal analyses were within-individual stratified Cox proportional hazards regressions, with each individual entering as a separate stratum in the analysis and serving as his/her own control. The obtained hazard ratio is thus adjusted for (i.e. stratified on) all potential time-invariant confounders within each individual. To adjust for age, which is a time-varying potential confounder, age was added to the model as a time-varying covariate, with one factor for each whole year. In the within-individual stratified Cox proportional hazards regression, only individuals who change medication status contribute directly to the estimate. All other individuals contribute indirectly through the estimates of other covariates. Since the covariates in the within-individual stratified Cox proportional hazards regression are time-varying, we did not test for the proportional hazards assumption. More information on this approach is provided here [32], and has been applied in studies of ADHD medication, antipsychotics and mood stabilisers [25-28]”.** |
|  |  | (*b*) Describe any methods used to examine subgroups and interactions  **In the Sensitivity Analyses on pages 9-10 we wrote:**  **“Finally, diuretics were used as an alternative exposure for convicted violent crimes to test the model.**  **For individuals who started SSRI treatment after being convicted of a violent crime, the number of days between the date of committing the crime and the start of SSRI treatment was calculated. To exclude the possibility of reverse causation, i.e. if committing a violent crime increased the probability of subsequent SSRI treatment, new within-individual stratified Cox proportional hazards regressions were carried out excluding all individuals who received SSRI treatment within 7, 14, 30 or 60 days, respectively, after committing a violent crime from these analysis.**  **Finally, the robustness of results was tested by undertaking four alternative analyses: First, a conditional Poisson regression examined how changes in medication exposure were associated with changes in violent crimes within the same person, thus adjusting for time-invariant confounders. Second, we repeated the main models with different treatment periods; (a) a treatment period was defined as a series of SSRI prescriptions with no more than three months between two consecutive prescriptions, and (b) with no more than four months between two consecutive prescriptions. Third, we tested for delayed onsets of action of SSRIs by setting the first day of medication to eight weeks after the first collected prescription starting each medication period. Fourth, we tested for SSRI discontinuation effects by extending the end of a treatment period to; (a) three weeks after the date of the last SSRI prescription in that treatment period, and (b) twelve weeks after the date of the last SSRI prescription in that treatment period, respectively”.**  ” |
|  |  | (*c*) Explain how missing data were addressed  **N/A** |
|  |  | (*d*) *Cohort study*—If applicable, explain how loss to follow-up was addressed  **In the Statistical Analyses on pages 6-7 we wrote:**  **“Individuals were followed from January 1, 2006 to December 31, 2009, and follow-up was adjusted for migration, periods in prison or institutional youth care, hospitalisation, and death through linkage to the Migration Register, Prison Register, Patient Register and Cause of Death Register. Unobservable time, i.e. time abroad, in prison, and in hospital, was removed (truncated) from the follow-up time. Time after hospital discharge, release from prison, or immigration, was added to the observable cohort again.”**  *Case-control study*—If applicable, explain how matching of cases and controls was addressed  *Cross-sectional study*—If applicable, describe analytical methods taking account of sampling strategy |
|  |  | (*e*) Describe any sensitivity analyses  **In the Sensitivity Analyses on pages 8-10 we wrote:**  **“In sensitivity analyses, within-individual stratified Cox proportional hazards regressions were carried with the following alternative outcomes; convictions for non-violent crimes, convictions for substance-related crimes, arrests for violent crimes, arrests for non-violent crimes, arrests for substance-related crimes, non-fatal injuries from accidents, alcohol intoxications, and psychiatric hospitalisations. Furthermore, each SSRI medication was analysed separately, and periods of using of two or more SSRI medications were excluded to adjust for switching effects between SSRI medications. Furthermore, all SSRIs were entered in the same model as covariates to adjust for concurrent use of other SSRIs. Analyses were also stratified on type of SSRI medication with suspicions of violent crimes as an alternative outcome. Additionally, other antidepressants (venlafaxine, duloxetine, tricyclics, heterocyclics, mirtazapine, moclobemide, and bupropion) were used as an alternative exposure for convicted violent crimes. Treatment periods for these antidepressants were defined in the same manner as SSRI treatment periods. Finally, diuretics were used as an alternative exposure for convicted violent crimes to test the model.**  **For individuals who started SSRI treatment after being convicted of a violent crime, the number of days between the date of committing the crime and the start of SSRI treatment was calculated. To exclude the possibility of reverse causation, i.e. if committing a violent crime increased the probability of subsequent SSRI treatment, new within-individual stratified Cox proportional hazards regressions were carried out excluding all individuals who received SSRI treatment within 7, 14, 30 or 60 days, respectively, after committing a violent crime from these analysis.**  **Finally, the robustness of results was tested by undertaking four alternative analyses: First, a conditional Poisson regression examined how changes in medication exposure were associated with changes in violent crimes within the same person, thus adjusting for time-invariant confounders. Second, we repeated the main models with different treatment periods; (a) a treatment period was defined as a series of SSRI prescriptions with no more than three months between two consecutive prescriptions, and (b) with no more than four months between two consecutive prescriptions. Third, we tested for delayed onsets of action of SSRIs by setting the first day of medication to eight weeks after the first collected prescription starting each medication period. Fourth, we tested for SSRI discontinuation effects by extending the end of a treatment period to; (a) three weeks after the date of the last SSRI prescription in that treatment period, and (b) twelve weeks after the date of the last SSRI prescription in that treatment period, respectively”.** |

Continued on next page

| Results | | |
| --- | --- | --- |
| Participants | 13* | (a) Report numbers of individuals at each stage of study—eg numbers potentially eligible, examined for eligibility, confirmed eligible, included in the study, completing follow-up, and analysed  **In the Methods on page 5 we wrote:**  **“In the total population of Sweden aged 15 and older in 2006 (n=7,917,854), and residing in Sweden during follow-up (January 1, 2006 to December 31, 2009), we identified 856,493 individuals who were prescribed SSRI treatment.”** |
|  |  | (b) Give reasons for non-participation at each stage  **N/A** |
|  |  | (c) Consider use of a flow diagram  **N/A** |
| Descriptive data | 14* | (a) Give characteristics of study participants (eg demographic, clinical, social) and information on exposures and potential confounders  **In the Results on page 11 we wrote:**  **“Of 7,917,854 individuals in the general population investigated, 856,493 (10.8%) were prescribed SSRIs during 2006-2009, or 14.1% of all women and 7.5% of all men in the investigated population (see Table 1 for background characteristics). Of those prescribed SSRIs, 9.9% were between age 15-24, 12.7% were between age 25-34, 16.5% were between age 35-44, 15.6% were between age 45-54, 15.5% were between age 55-64, and 29.7% were age 65 and over at baseline in 2006. In the SSRI cohort, 8377 persons (1.0%) were convicted of a violent crime during 2006-2009”.**  **Please see Table 1 on pages 29-31 for more details on participants, exposures and outcomes.** |
|  |  | (b) Indicate number of participants with missing data for each variable of interest  **N/A** |
|  |  | (c) *Cohort study*—Summarise follow-up time (eg, average and total amount)  **In the Statistical Analyses on pages 6-7 we wrote:**  **“Individuals were followed from January 1, 2006 to December 31, 2009, and follow-up was adjusted for migration, periods in prison or institutional youth care, hospitalisation, and death through linkage to the Migration Register, Prison Register, Patient Register and Cause of Death Register.”** |
| Outcome data | 15* | *Cohort study*—Report numbers of outcome events or summary measures over time  **Please see the last column (labelled ‘no of events’) in Tables 2-4 on pages 32-42.** |
|  |  | *Case-control study—*Report numbers in each exposure category, or summary measures of exposure |
|  |  | *Cross-sectional study—*Report numbers of outcome events or summary measures |
| Main results | 16 | (*a*) Give unadjusted estimates and, if applicable, confounder-adjusted estimates and their precision (eg, 95% confidence interval). Make clear which confounders were adjusted for and why they were included  **In the Results on page 11 we wrote:**  **“Within-individual Cox proportional analyses were carried out to compare violent crime rates within the same individual when they were on medication compared to periods when they were not, and results showed increased risks of violent crime convictions during medicated periods (hazard ratio [HR]=1.19; 95% CI 1.08-1.32; p-value=0.0005; Table 2). To ensure adherence to treatment, we excluded individuals with only one collected prescription, resulting in a similar hazard (HR=1.22, 1.10-1.35, p=0.0001). To test if individuals who had collected one prescription only differed from the rest of the cohort, we also carried out a within-individual analysis including these individuals only. No significant associations with convicted violent crimes were found for this group (HR=0.73, 0.45-1.17, p=0.1933). After adjustment for concurrent psychotropic medications, the estimated hazard did not materially change (HR=1.22, 1.11-1.32, p=0.0005). Additionally, when we excluded all individuals who had received other psychotropic medications during follow-up from the analysis, the estimated hazard was similar (HR=1.20, 1.04-1.38, p=0.0144). The between-individual Cox proportional analysis also demonstrated an association between SSRI prescriptions and being convicted of a violent crime (HR=2.66, 2.54-2.78, p=0.0001) when comparing individuals on SSRIs to individuals who were not taking SSRIs.”** |
|  |  | (*b*) Report category boundaries when continuous variables were categorized  **In the Statistical Analyses on page 8 we wrote:**  **“To estimate cumulative exposure of SSRIs, the defined daily dose (DDD) of SSRI medication [30] was calculated through summing collected medication, and then dividing the sum by the number of days in the treatment period. DDDs were categorized into 4 groups; (1) no exposure; (2) low SSRI exposure (<1 DDD/day); (3) moderate SSRI exposure (1-2 DDD/day), and; (4) high SSRI exposure (>2 DDD/day).”** |
|  |  | (*c*) If relevant, consider translating estimates of relative risk into absolute risk for a meaningful time period  **N/A** |
| Other analyses | 17 | Report other analyses done—eg analyses of subgroups and interactions, and sensitivity analyses  **In the Results on pages 12-14 we wrote:**  **“In sensitivity analyses, the relationship between SSRI treatment and other outcomes was examined (Table 4), and results showed an increased risk of violent arrests (HR=1.13, 1.08-1.18, p=0.0001), and non-violent convictions (HR=1.10, 1.05-1.15, p=0.0001) and arrests (HR=1.05, 1.02-1.08, p=0.0004). Furthermore, an increased risk of non-fatal injuries from accidents was shown (HR=1.20, 1.18-1.23, p=0.0001). The possible role of alcohol as a time-varying confounder was tested by using hospitalisations for alcohol intoxication as an outcome, showing an increased risk during times of medication (HR=1.06, 1.03-1.09, p=0.0001). The risk of hospitalisations in psychiatric care was also examined, showing a slightly decreased risk (HR=0.96, 0.93-0.99, p=0.0119). When we investigated other antidepressant classes, we found significant associations with convicted violent crimes for individuals prescribed venlafaxine (HR=1.39, 1.10-1.78, p=0.0071). The risk of being convicted of a violent crime was reduced when on mirtazapine (HR=0.71, 0.59-0.88, p=0.0011). Finally, associations between convicted violent crimes and diuretics were carried out as a negative control analysis of the within-individual model, and results showed that the risk was reduced when on diuretics (HR=0.80, 0.67-0.95, p=0.0121).**  **When stratifying all analyses on age (Appendix Table 1), the increased risk of being convicted of a violent crime remained in ages 15 to 24 after adjustment for concurrent psychotropic medications (HR=1.45, 1.21-1.74, p=0.0001). Results also showed that low SSRI exposure was associated with an increased risk of being convicted of a violent crime in this age-band only (HR=1.62, 1.23-2.13, p=0.0006). Furthermore, significant associations were shown for violent arrests, and suspected and convicted non-violent crimes for ages 15 to 24, but also for ages 25 to 34, although associations were weaker in the latter age-band. The increased risk of non-fatal injuries from accidents remained significant for all ages. Results also showed that individuals aged 15-24 and 25-34 had an increased risk of being hospitalized due to alcohol intoxication (HR=1.98, 1.76-2.21, p=0.0001, HR=1.33, 1.21-1.46, p=0.0001). However, individuals aged 45 and older showed a slightly decreased risk of being hospitalized for alcohol intoxication (HR=0.96, 0.93-0.99, p=0.0276).**  **To account for the possibility of reverse causation, i.e. that individuals were taking SSRIs after committing a crime, we excluded 996 individuals who received SSRIs within 60 days of committing a violent crime, and the risk increase remained (HR=1.14, 1.01-1.28, p=0.0388), We then excluded 608 individuals who received SSRIs within 30 days of committing of a violent crime, and the risk increase remained similar (HR=1.15, 1.03-1.29, p=0.0153). When we excluded those who received medication within 14 days (356 individuals) or 7 days (197 individuals) of committing a violent crime, similar risk increases were found (Table 4). No material differences were found when we repeated this with suspicions of a violent crime as an outcome (Appendix Table 2). When we carried out a conditional Poisson regression, a similar pattern of findings was found (incidence rate ratio [IRR] for violent crime=1.18, 1.09-1.27, p=0.000). When defining treatment periods as no breaks of at least 3 or 4 months, respectively, in within-individual models, no material differences were found (Appendix Table 2). When testing for delayed treatment effects, no material differences were found 8 weeks after starting SSRI treatment (HR=1.21, 1.02-1.43, p=0.0311). Similar effects were found when testing for SSRI discontinuation effects up to three weeks or twelve weeks, respectively, after the last collected prescription (Appendix Table 2).”** |
| Discussion | | |
| Key results | 18 | Summarise key results with reference to study objectives  **Objectives:**  **Our objective was to investigate the association between SSRIs and violence outcomes by linking data from Swedish national registers on individual SSRIs prescriptions, use of other psychotropic drugs, violent crimes, and alternative outcomes in a large population-based cohort.**  **Key results:**  **There was an overall association between SSRIs and violent convictions (HR=1.19, 95% CI: 1.08-1.32, p-value =0.0005). On age stratification, there was a significant association between SSRIs and violent convictions for individuals aged 15 to 24 (HR=1.43, 1.19-1.73, p=0.0002). However, there were no significant associations in those aged 25-34 (HR=1.20, 0.95-1.52, p=0.1252), in those aged 35-44 (HR=1.06, 0.83-1.35, p=0.6662) or in those older than 45 (HR=1.07, 0.84-1.35, p=0.1403). Associations in those aged 15 to 24 were also found for violent arrests (HR=1.28, 1.16-1.41, p=0.0001), non-violent convictions (HR=1.22, 1.10-1.34, p=0.0001) and arrests (HR=1.13, 1.07-1.20, p=0.0001), and non-fatal hospitalized accidents (HR=1.29, 1.22-1.36, p=0.0001).** |
| Limitations | 19 | Discuss limitations of the study, taking into account sources of potential bias or imprecision. Discuss both direction and magnitude of any potential bias  **In the Discussion on pages 19-21 we wrote:**  **“Limitations included the use of diagnoses from the Patient Register, which only includes diagnoses from specialists. Also, the use of official sources of data for crime outcomes is likely to underestimate true rates and possibly involve selection effects. However, we tried to address such biases by using arrests (as opposed to convictions) and also by examining accidents. It is not clear whether these findings will translate into less severe forms of violence or those not reported to the police, and triangulating the findings with information from self or informant-reported violence will be an important future research question. Another limitation was that detailed information about the actual prescriptions was not available. Although our data are an improvement on prescription data as they include prescriptions that are dispensed by pharmacies and collected by individuals, we were unable to account for lack of, or variations in, adherence. This problem is parallel to non-adherence in randomised controlled trials, and our within-individual estimate is comparable to the intention-to-treat analysis used in randomised controlled trials. If individuals consumed SSRIs during periods when we assumed that there were not, then this should reduce the hazards reported and suggest that our estimates are underestimates. A possible source of underestimation is that we excluded persons who were prescribed SSRIs on one occasion, and they may have discontinued the medications due to adverse effects that we did not measure. We thus carried out analyses where individuals with a single prescription were included, and found no material differences in hazards. Another is that we used a conservative approach to measure the end of a treatment period (we defined this as the date of the last SSRI prescription in a treatment period), which could result in slightly lower sensitivity (i.e. individuals classified as unmedicated when truly medicated). However, sensitivity analyses using less conservative approaches to measure the end of a treatment period (three weeks and twelve weeks, respectively, after the last collected SSRI prescription in a treatment period) resulted in similarly increased risks of violent crimes. Sweden has higher prescription rates of SSRIs than the average for Europe (5-year-mean DDD/1000/day: Sweden=70.1; across 29 European countries=40.0) [14], and similar to the US (10.8% treated in our cohort between 2006-2009 vs. 10.1% treated in the US in 2005) [55]. In relation to criminality, Sweden has similar police-reported assault rates as compared to the US [56]. Finally, there might be residual confounding for the within-individual estimates due to unmeasured time-varying confounders. However, we are not aware of any statistical method that also allows adjustment for unmeasured time-varying confounders.”** |
| Interpretation | 20 | Give a cautious overall interpretation of results considering objectives, limitations, multiplicity of analyses, results from similar studies, and other relevant evidence  **In the Discussion on page 19 we wrote:**  **“There are two principal clinical implications arising from this study. First, no associations between SSRIs and violent crimes were found for the majority of people who were prescribed these medications, including all persons aged 25 years and older. Second, the risk increase we report in young people is not insignificant, and hence warrants further examination. If our findings related to young people are validated in other designs, samples, and settings, warnings about increased risks of violent behaviours while being treated with SSRIs may be needed. Any such changes will need to be carefully considered, as the public health benefit from decreases in violence following restrictions in SSRI use may be countered by increases in other adverse outcomes (such as more disability, rehospitalisation, or suicides). From a public health perspective, this might argue against restrictions on the primary care prescribing of SSRIs as long as potential risks are disclosed”.** |
| Generalisability | 21 | Discuss the generalisability (external validity) of the study results  **In the Discussion on pages 20-21 we wrote:**  **“Sweden has higher prescription rates of SSRIs than the average for Europe (5-year-mean DDD/1000/day: Sweden=70.1; across 29 European countries=40.0) [14], and similar to the US (10.8% treated in our cohort between 2006-2009 vs. 10.1% treated in the US in 2005) [55]. In relation to criminality, Sweden has similar police-reported assault rates as compared to the US [56].”** |
| Other information | | |
| Funding | 22 | Give the source of funding and the role of the funders for the present study and, if applicable, for the original study on which the present article is based  **Dr. Molero and Dr. Hellner Gumpert are supported by Karolinska Institutet. Mr. Zetterqvist and Prof. Lichtenstein are supported by grants from the Swedish Research Council. Prof. Fazel is supported by the Wellcome Trust [095806].**  **Funders had no further role in the study design, in the collection of data or in the analysis and interpretation of data.** |

*Give information separately for cases and controls in case-control studies and, if applicable, for exposed and unexposed groups in cohort and cross-sectional studies.

**Note:** An Explanation and Elaboration article discusses each checklist item and gives methodological background and published examples of transparent reporting. The STROBE checklist is best used in conjunction with this article (freely available on the Web sites of PLoS Medicine at http://www.plosmedicine.org/, Annals of Internal Medicine at http://www.annals.org/, and Epidemiology at http://www.epidem.com/). Information on the STROBE Initiative is available at www.strobe-statement.org.
