# Supplementary material for: Selective Serotonin Reuptake Inhibitors and Violent Crime: A Cohort Study
Source: PLoS Med. 2015 Sep 15;12(9):e1001875. doi: 10.1371/journal.pmed.1001875 (PMC4570770; doi:10.1371/journal.pmed.1001875)
Supplement: S1 Methods — (DOCX) [file pmed.1001875.s002.docx]

**S1 Methods**

**SSRI medications**

Among the 856,493 individuals who were prescribed SSRI treatment, 65,862 individuals were prescribed fluoxetine (N06AB03), 389,857 citalopram (N06AB04), 46,615 paroxetine (N06AB05), 215,873 sertraline (N06AB06), 1198 fluvoxamine (N06AB08), and 84,934 individuals were prescribed escitalopram (N06AB10).

**Crimes**

Information on convictions for violent crimes included attempted, completed and aggravated forms of: homicide, manslaughter, unlawful threats, harassment, robbery, arson, assault, assault on an official, kidnapping, stalking, coercion, and all sexual offences, and the date of perpetration was identified for each conviction. Convictions for substance-related crimes included manufacturing alcohol, driving under the influence of alcohol or illicit substances, smuggling illicit substances, manufacturing illicit substances, supplying illicit substances, possession of illicit substances, and personal use of illicit substances. Convictions for non-violent crimes included all offences other than violent crimes and substance-related crimes. Convictions were extracted from the National Crime Register, including all convictions in Swedish district courts [1].

Conviction data were used because individuals are convicted as guilty regardless of mental illness in Sweden, thus conviction data included persons who received custodial or noncustodial sentences and individuals transferred to forensic hospital (e.g., individuals who were deemed to have suffered from severe mental disorder at the time of the offense). Furthermore, conviction data also included those cases in which the prosecutor decided to caution or fine (e.g., less serious sexual crimes and some juvenile cases). In addition, though sentencing decisions may vary according to background factors, plea-bargaining at the conviction stage is not part of the Swedish legal system. Therefore, conviction data more accurately reflect the extent of officially resolved criminality in the population. The crime register has total national coverage—only 0.05% of all registered convictions had incomplete personal identification numbers in another study [2].

Arrests, as distinct to convictions, were identified in the Register of Persons Suspected of Offenses, which includes all individuals suspected of crime after a completed investigation by police, the customs authority, or the prosecution service [2], regardless of whether suspicion leads to prosecution or conviction.

**Other psychotropic medications**

Adjustments were made for concurrent psychotropic medications other than SSRIs, and included: Antipsychotics (N05A, except N05AN01), hypnotics, sedatives and anxiolytics (N05B and N05C), drugs used in addictive disorders (N07B), mood stabilizers (N03AF01, N03AF02, N03AG01, N05AN01, N03AX09), antiepileptics (N03A, except mood stabilizers), and antidepressant medications other than SSRIs (venlafaxine [N06AX16], duloxetine [N06AX21], tricyclics [N06AA04, N06AA09, N06AA10], heterocyclics [N06AA21, N06AX03], mirtazapine [N06AZ11], non-selective monoamine oxidase inhibitors [N06AF], moclobemide [N06AG02], bupropion [N06AX12]). Antidepressant medications other than SSRIs were also used as alternative exposure in sensitivity analyses. Information on medication and date of the collected prescription was extracted from the Prescribed Drug Register [3].

Non-psychotropic medications

In further sensitivity analyses, diuretics (C03) were used as an alternative exposure. Information was collected from the Prescribed Drug Register [3].

**Psychiatric diagnoses**

Psychiatric diagnoses were defined as: psychotic disorders (ICD-8: 295, 297-299, ICD-9: 295, 297, 298, ICD-10: F20-F29), mood disorders (ICD-8: 296, 300.4, ICD-9: 296, 300E, 311, ICD-10: F30-F39), anxiety, dissociative, stress-related, and somatoform disorders (ICD-8: 300 except 300.4, 307, ICD-9: 300 except 300.E, 308-309, ICD-10: F40-F45, F48, 6), eating disorders (ICD-9: 307B, 307F, ICD-10: F50), and psychoactive substance misuse (ICD-8: 291, 303, 304, ICD-9: 291, 303, 304, 305A, 305X, ICD-10: F10-F19). Diagnoses were collected from the patient register [4], which includes diagnoses from both hospitalisations and outpatient visits in specialised care.

**Non-fatal injuries from accidents**

These were defined as emergency inpatient or outpatient episodes of: transport accidents (ICD10: V01-V99), other external causes of accidental injury (ICD10: W00-W99, X00-X49, X58-X59), sequelae of external causes of morbidity and mortality (ICD10: Y85, Y86), supplementary factors related to morbidity and mortality classified elsewhere (ICD10: Y90-Y98), and injury and certain other consequences of external causes, excluding poisoning (ICD10: S00-S99, T00-T35, T90-T94). Diagnoses were collected from the patient register [4]. Diagnoses received during planned visits (i.e. follow-ups and referrals) were excluded from the analyses. Although this is a more conservative estimate, this measure was used to avoid overestimation of diagnoses, as the diagnosis that is the reason for treatment initiation is also coded during follow-ups and referrals.

**Alcohol intoxications and emergency alcohol misuse**

Alcohol intoxications and emergency alcohol misuse were defined as emergency inpatient or outpatient episodes of mental and behavioural disorders due to the use of alcohol (ICD10: F10), toxic effect of alcohol (ICD10: T51), and supplementary factors; evidence of alcohol involvement determined by blood alcohol level (ICD10: Y90) or by level of intoxication (ICD10: Y91). Diagnoses were collected from the patient register [4], and diagnoses received during planned visits (i.e. follow-ups and referrals) were excluded from the analyses.

**Psychiatric hospitalisations**

Information on emergency inpatient or outpatient episodes in psychiatric care was extracted from the patient register [4], and included care in general psychiatry, child and adolescent psychiatry, geriatric psychiatry and forensic psychiatry. Planned visits (i.e. follow-ups and referrals) in psychiatric care were excluded. Because of the risk of reverse causation between SSRIs and psychiatric hospitalisations (i.e. being treated at a psychiatric hospital increases the probability of subsequent SSRI treatment), the first eight first weeks of SSRI treatment were removed when analysing of this outcome.

**REFERENCES**

1. National Council for Crime Prevention. Kriminalstatistik 2010 [Criminal Statistics 2010]. Västerås: National Council for Crime Prevention, 2010.

2. Fazel S, Grann M. The population impact of severe mental illness on violent crime. Am J Psychiatry. 2006;163(8):1397-1403.

3. Wettermark B, Hammar N, MichaelFored C, Leimanis A, Otterblad Olausson P, Bergman U et al. The new Swedish Prescribed Drug Register – Opportunities for pharmacoepidemiological research and experience from the first six months. Pharmacoepidem Drug Safety. 2007;16(7):726–735.

4. Ludvigsson J, Andersson E, Ekbom A, Feychting M, Kim JL, Reuterwall C et al. External review and validation of the Swedish national inpatient register. BMC Public Health. 2011;11:450.
