# Supplementary material for: Selective Serotonin Reuptake Inhibitors and Violent Crime: A Cohort Study
Source: PLoS Med. 2015 Sep 15;12(9):e1001875. doi: 10.1371/journal.pmed.1001875 (PMC4570770; doi:10.1371/journal.pmed.1001875)
Supplement: S1 Table — (DOCX) [file pmed.1001875.s003.docx]

**S1 TABLE:** Rates of being convicted of a violent crime and alternative outcomes in individuals treated with SSRI medication compared to non-treatment periods in the same person, stratified on age using stratified Cox regression models.

|  | **Hazard ratio**  **(95 % CI)** | **P-value** |
| --- | --- | --- |
| **All individuals adjusted for other psychotropic medications** | | |
| 15 to 24 | 1.45 (1.21-1.74) | 0.000 |
| 25 to 34 | 1.21 (0.97-1.52) | 0.088 |
| 35 to 44 | 0.99 (0.78-1.25) | 0.925 |
| 45 and over | 1.10 (0.88-1.38) | 0.398 |
|  | | |
| **Excluding individuals with other psychotropic medications during follow-up** | | |
| 15 to 24 | 1.35 (1.06-1.71) | 0.015 |
| 25 to 34 | 1.16 (0.83-1.63) | 0.395 |
| 35 to 44 | 0.86 (0.59-1.25) | 0.431 |
| 45 and over | 1.10 (0.77-1.57) | 0.584 |
|  | | |
| **Low SSRI exposure** | | |
| 15 to 24 | 1.62 (1.23-2.13) | <0.001 |
| 25 to 34 | 1.32 (0.95-1.83) | 0.098 |
| 35 to 44 | 1.26 (0.89-1.78) | 0.192 |
| 45 and over | 0.92 (0.67-1.26) | 0.599 |
|  | | |
| **Alternative outcomes** | | |
| **Convicted of a non-violent crime** | | |
| 15 to 24 | 1.22 (1.10-1.34) | <0.001 |
| 25 to 34 | 1.18 (1.07-1.28) | <0.001 |
| 35 to 44 | 0.96 (0.87-1.05) | 0.376 |
| 45 and over | 1.05 (0.98-1.13) | 0.191 |
|  | | |
| **Convicted of a substance-related crime** |  |  |
| 15 to 24 | 1.15 (1.01-1.32) | 0.039 |
| 25 to 34 | 1.01 (0.89-1.15) | 0.871 |
| 35 to 44 | 0.91 (0.77-1.06) | 0.214 |
| 45 and over | 0.95 (0.81-1.11) | 0.510 |
|  | | |
| **Suspected of a violent crime** | | |
| 15 to 24 | 1.28 (1.16-1.41) | <0.001 |
| 25 to 34 | 1.15 (1.04-1.28) | 0.007 |
| 35 to 44 | 1.04 (0.94-1.14) | 0.452 |
| 45 and over | 1.00 (0.91-1.10) | 0.968 |
|  | | |
| **Suspected of a non-violent crime** | | |
| 15 to 24 | 1.13 (1.07-1.20) | <0.001 |
| 25 to 34 | 1.06 (1.00-1.11) | 0.044 |
| 35 to 44 | 0.97 (0.92-1.03) | 0.378 |
| 45 and over | 1.04 (0.98-1.09) | 0.220 |
|  | | |
| **Suspected of a substance-related crime** | | |
| 15 to 24 | 1.08 (0.96-1.21) | 0.214 |
| 25 to 34 | 1.00 (0.90-1.11) | 0.960 |
| 35 to 44 | 0.98 (0.86-1-12) | 0.755 |
| 45 and over | 0.90 (0.79-1.03) | 0.113 |
|  | | |
| **Non-fatal injuries from accidents** | | |
| 15 to 24 | 1.29 (1.22-1.36) | <0.001 |
| 25 to 34 | 1.21 (1.13-1.29) | <0.001 |
| 35 to 44 | 1.11 (1.04-1.17) | 0.001 |
| 45 and over | 1.17 (1.14-1.19) | <0.001 |
|  | | |
| **Alcohol intoxication** | | |
| 15 to 24 | 1.98 (1.76-2.21) | <0.001 |
| 25 to 34 | 1.33 (1.21-1.46) | <0.001 |
| 35 to 44 | 1.08 (1.01-1.14) | 0.015 |
| 45 and over | 0.96 (0.93-0.99) | 0.028 |
|  | | |
| **Type of SSRI medication and convicted violent crimes as outcome** | | |
| **Fluoxetine** | | |
| 15 to 24 | 0.81 (0.52-1.25) | 0.338 |
| 25 to 34 | 1.57 (0.80-3.07) | 0.191 |
| 35 to 44 | 1.22 (0.46-3.25) | 0.684 |
| 45 and over | 1.32 (0.70-2.52) | 0.391 |
|  | | |
| **Citalopram** | | |
| 15 to 24 | 1.31 (0.93-1.86) | 0.126 |
| 25 to 34 | 1.34 (0.93-1.96) | 0.115 |
| 35 to 44 | 1.12 (0.73-1.71) | 0.616 |
| 45 and over | 0.85 (0.60-1.21) | 0.367 |
|  | | |
| **Paroxetine** | | |
| 15 to 24 | 0.59 (0.25-1-41) | 0.234 |
| 25 to 34 | 0.98 (0.45-2.13) | 0.958 |
| 35 to 44 | 1.70 (0.85-3.40) | 0.130 |
| 45 and over | 1.37 (0.61-3.06) | 0.442 |
|  | | |
| **Sertraline** | | |
| 15 to 24 | 1.66 (1.21-2.29) | 0.001 |
| 25 to 34 | 0.91 (0.59-1.42) | 0.687 |
| 35 to 44 | 0.91 (0.61-1.35) | 0.617 |
| 45 and over | 1.23 (0.78-1.95) | 0.370 |
|  | | |
| **Escitalopram** | | |
| 15 to 24 | 2.44 (1.40-4.25) | 0.001 |
| 25 to 34 | 1.28 (0.71-2.34) | 0.417 |
| 35 to 44 | 0.54 (0.28-1.03) | 0.060 |
| 45 and over | 0.80 (0.44-1.44) | 0.450 |
|  | | |
| **Type of SSRI medication and suspected violent crimes as outcome** | | |
| **Fluoxetine** | | |
| 15 to 24 | 1.08 (0.86-1.36) | 0.487 |
| 25 to 34 | 0.91 (0.68-1.23) | 0.554 |
| 35 to 44 | 0.86 (0.64-1.16) | 0.315 |
| 45 and over | 0.98 (0.74-1.31) | 0.899 |
|  | | |
| **Citalopram** | | |
| 15 to 24 | 1.45 (1.20-1.75) | 0.000 |
| 25 to 34 | 1.26 (1.06-1.50) | 0.008 |
| 35 to 44 | 1.11 (0.95-1.30) | 0.206 |
| 45 and over | 1.00 (0.87-1.15) | 0.970 |
|  | | |
| **Paroxetine** | | |
| 15 to 24 | 1.40 (0.90-2.16) | 0.131 |
| 25 to 34 | 1.31 (0.89-1.92) | 0.171 |
| 35 to 44 | 1.29 (0.96-1.72) | 0.094 |
| 45 and over | 0.96 (0.72-1.29) | 0.799 |
|  | | |
| **Sertraline** | | |
| 15 to 24 | 1.12 (0.95-1.32) | 0.183 |
| 25 to 34 | 1.10 (0.92-1.32) | 0.307 |
| 35 to 44 | 0.94 (0.80-1.10) | 0.441 |
| 45 and over | 0.97 (0.80-1.16) | 0.710 |
|  | | |
| **Escitalopram** | | |
| 15 to 24 | 1.32 (0.98-1.77) | 0.067 |
| 25 to 34 | 1.12 (0.85-1.47) | 0.421 |
| 35 to 44 | 0.95 (0.73-1.24) | 0.707 |
| 45 and over | 1.02 (0.80-1.31) | 0.861 |
|  | | |
| **Other antidepressants and convicted violent crimes as outcome** | | |
| **Venlafaxine** | | |
| 15 to 24 | 2.46 (1.33-4.54) | 0.004 |
| 25 to 34 | 1.55 (0.93-2.57) | 0.094 |
| 35 to 44 | 1.38 (0.83-2.30) | 0.224 |
| 45 and over | 0.71(0.45-1.14) | 0.157 |
|  | | |
| **Duloxetine** | | |
| 15 to 24 | 2.12 (0.99-4.58) | 0.054 |
| 25 to 34 | 1.26 (0.56-2.80) | 0.579 |
| 35 to 44 | 0.77 (0.32-1.85) | 0.555 |
| 45 and over | 1.19 (0.62-2.28) | 0.606 |
|  | | |
| **Tricyclics** | | |
| 15 to 24 | 1.20 (0.30-4.89) | 0.795 |
| 25 to 34 | 2.35 (1.04-5.32) | 0.041 |
| 35 to 44 | 0.71 (0.30-1.67) | 0.432 |
| 45 and over | 0.97 (0.52-1.83) | 0.923 |
|  | | |
| **Heterocyclics** | | |
| 15 to 24 | 2.94 (0.77-11.16) | 0.113 |
| 25 to 34 | 3.19 (0.69-14.75) | 0.137 |
| 35 to 44 | 2.31 (0.64-8.33) | 0.201 |
| 45 and over | 0.70 (0.24-2.07) | 0.523 |
|  | | |
| **Mirtazapine** | | |
| 15 to 24 | 0.58 (0.37-0.90) | 0.014 |
| 25 to 34 | 0.90 (0.58-1.27) | 0.436 |
| 35 to 44 | 0.90 (0.59-1.37) | 0.630 |
| 45 and over | 0.67 (0.42-1.07) | 0.091 |
|  | | |

Note: Analyses excluded individuals with only one collected prescription.

Note: Too few individuals for age-band analyses on fluvoxamine, monoamine oxidase inhibitors, moclobemide and bupropion
