# Supplementary material for: Selective Serotonin Reuptake Inhibitors and Violent Crime: A Cohort Study
Source: PLoS Med. 2015 Sep 15;12(9):e1001875. doi: 10.1371/journal.pmed.1001875 (PMC4570770; doi:10.1371/journal.pmed.1001875)
Supplement: S2 Table — (DOCX) [file pmed.1001875.s004.docx]

**S2 TABLE:** Rates of being convicted and suspected of a violent crime in individuals treated with SSRI medication compared to non-treatment periods in the same person using Conditional Poisson regression, stratified regression Cox models for alternative treatment periods, and when excluding individuals who received SSRIs after committing a crime.

|  | | **Incidence Rate Ratio**  **(95 % CI)** | | **P-value** |
| --- | --- | --- | --- | --- |
| **Conditional Poisson regression** | | 1.18 (1.09-1.27) | | 0.001 |
|  | | | | |
|  | | **Hazard ratio**  **(95 % CI)** | | **P-value** |
| **Alternative treatment periods** | | | | |
| At least two collected prescriptions  within a 3-month period | | 1.18 (1.05-1.33) | | 0.004 |
| At least two collected prescriptions  within a 4-month period | | 1.13 (1.02-1.26) | | 0.021 |
| Excluding the 8 first weeks of  treatment | | 1.21 (1.02-1.43) | | 0.031 |
| Treatment until 3 weeks after last  collected prescription | | 1.21 (1.09-1.35) | | 0.000 |
| Treatment until 12 weeks after last  collected prescription | | 1.22 (1.10-1.35) | | 0.000 |
|  | | | | |
| **Exclusion of individuals who received SSRIs after committing a violent crime: convicted crime as outcome** | | | | |
| Excluding individuals who received  SSRIs within 60 days | | 1.14 (1.01-1.28) | | 0.038 |
| Excluding individuals who received  SSRIs within 30 days | | 1.15 (1.03-1.29) | | 0.015 |
| Excluding individuals who received  SSRIs within 14 days | | 1.17 (1.05-1.30) | | 0.006 |
| Excluding individuals who received  SSRIs within 7 days | | 1.20 (1.07-1.33) | | 0.001 |
|  | | | | |
| **Exclusion of individuals who received SSRIs after committing a violent crime: suspected crime as outcome** | | | | |
| Excluding individuals who  received SSRIs within 60 days | 1.11 (0.98-1.26) | | 0.089 | |
| Excluding individuals who  received SSRIs within 30 days | 1.14 (1.01-1.28) | | 0.031 | |
| Excluding individuals who  received SSRIs within 14 days | 1.16 (1.04-1.29) | | 0.009 | |
| Excluding individuals who  received SSRIs within 7 days | 1.18 (1.06-1.32) | | 0.002 | |
